# Non-genomic effects of the Pregnane X Receptor negatively regulate platelet functions, thrombosis and haemostasis

**DOI:** 10.1038/s41598-019-53218-x

**Published:** 2019-11-20

**Authors:** Gagan D. Flora, Khaled A. Sahli, Parvathy Sasikumar, Lisa-Marie Holbrook, Alexander R. Stainer, Sarah K. AlOuda, Marilena Crescente, Tanya Sage, Amanda J. Unsworth, Jonathan M. Gibbins

**Affiliations:** 1 Institute for Cardiovascular and Metabolic Research, School of Biological Sciences, University of Reading, Reading, UK; 2 Department of Internal Medicine, University of Iowa, Iowa City, IA USA; 3 General Directorate of Medical Services, Ministry of Interior, Riyadh, Kingdom of Saudi Arabia; 4 Centre for Haematology, Imperial College London, London, UK; 5 School of Cardiovascular Medicine and Sciences, King’s College London, London, UK; 6 Centre for Immunobiology, Blizard Institute, Barts and The London School of Medicine and Dentistry, Queen Mary University of London, London, UK; 7 School of Healthcare Science, Manchester Metropolitan University, Manchester, UK

**Keywords:** Nuclear receptors, Mechanisms of disease

## Abstract

The pregnane X receptor (PXR) is a nuclear receptor (NR), involved in the detoxification of xenobiotic compounds. Recently, its presence was reported in the human vasculature and its ligands were proposed to exhibit anti-atherosclerotic effects. Since platelets contribute towards the development of atherosclerosis and possess numerous NRs, we investigated the expression of PXR in platelets along with the ability of its ligands to modulate platelet activation. The expression of PXR in human platelets was confirmed using immunoprecipitation analysis. Treatment with PXR ligands was found to inhibit platelet functions stimulated by a range of agonists, with platelet aggregation, granule secretion, adhesion and spreading on fibrinogen all attenuated along with a reduction in thrombus formation (both *in vitro* and *in vivo*). The effects of PXR ligands were observed in a species-specific manner, and the human-specific ligand, SR12813, was observed to attenuate thrombus formation *in vivo* in humanised PXR transgenic mice. PXR ligand-mediated inhibition of platelet function was found to be associated with the inhibition of Src-family kinases (SFKs). This study identifies acute, non-genomic regulatory effects of PXR ligands on platelet function and thrombus formation. In combination with the emerging anti-atherosclerotic properties of PXR ligands, these anti-thrombotic effects may provide additional cardio-protective benefits.

## Introduction

Nuclear receptors (NRs) are well characterised for their genomic functions (transcriptional regulation), however, less is known about their non-genomic roles. Several NRs (such as LXR, PPARα/β/γ, RXR, RAR and FXR) have been identified in platelets, which upon ligation regulate platelet activity, thrombosis and haemostasis through a variety of mechanisms in a non-genomic manner^1–7^. The pregnane X receptor (PXR) forms a heterodimer with retinoid X receptor (RXR)^8^ and functions as a sensor, activated by xenobiotic and toxic endogenous compounds, leading to their metabolism and elimination through the upregulation of cytochrome P450 enzymes. PXR is predominantly expressed in liver and intestines^9^ and unlike other NRs, displays a large and flexible ligand-binding domain (LBD) that enables its binding with a diverse array of ligands that includes bile acids, pharmaceutical substrates, herbal medicines, environmental pollutants, and endobiotics^10^. Additionally, the LBD of PXR shows variation in amino acid sequence amongst different species^11^. Therefore, inter-species differences in the ligands that activate PXR have been reported. Consequently, human PXR is activated by ligands such as SR12813 and rifampicin, whilst they do not affect mouse PXR. Similarly, the PXR ligand, pregnenolone 16α-carbonitrile (PCN), is highly specific to rodents only^12,13^.

Increasing evidence identifies PXR to act as a potential therapeutic target for the treatment of a variety of patho-physiologies^14^. PXR ligands have demonstrated anti-atherosclerotic effects via increased cholesterol clearance and HDL production in a mouse model of atherosclerosis^15–17^. Recently, PXR was reported to be expressed in the human vasculature (blood vessels, aortic endothelial and smooth muscle cells), where it functions to detoxify circulating toxins and avert vascular damage by upregulating CYP 3A, 2B and 2C activity^18^.

Here, we report the presence of PXR in human platelets. Treatment of platelets with PXR ligands (SR12813 or rifampicin) attenuated platelet functions and thrombus formation (*in vitro* and *in vivo*) through a mechanism that is associated with the down-regulation of Src-family kinase (SFK) signalling.

## Materials and Methods

### Reagents

Bovine thrombin, rifampicin and 5-Pregnen-3β-ol-20-one-16α-carbonitrile (PCN) were purchased from Sigma-Aldrich. Collagen was obtained from Nycomed. CRP-XL was from Professor R. Farndale (University of Cambridge). SR12813 was from Abcam (Cambridge, UK). Primary polyclonal anti-PXR (sc25381), monoclonal anti-RXRα/β/γ (sc46659), 14-3-3 ζ (sc-293415) and actin (sc-1615) antibodies were from SantaCruz. Monoclonal anti-PXR (ab41930), primary phospho anti-Lyn (Y396) (ab226778), Syk (Y525/526) (ab58575) and LAT (Y200) (ab68139) antibodies were from Abcam. Primary phospho anti-Src (Y418) (#44-660 G) was from ThermoFisher Scientific. Primary phospho anti-PLCγ2 (Y1217) (#3871), VASP (S157 and S239) (#3111 and #3114) and PKC (#2261) were purchased from Cell Signalling Technologies. Anti-phospho-Tyr 4G10 (#05-321) antibody was from Millipore. Fluorophore conjugated secondary antibodies, Fura-2AM and Alexa-488 conjugated phalloidin were from Life Technologies. PE-Cy5 anti-CD62P antibody was from BD Biosciences. FITC-labelled anti-fibrinogen was from Dako. All other reagents were from previously described sources^5,6^. Humanised PXR transgenic mice [C57BL/6-Nr1i2^tm1(NR1I2)Arte^] were purchased from Taconic Biosciences (Denmank) and bred under licence at the bioresource unit of the University of Reading.

### Human blood collection

Blood was collected with approval by the University of Reading Research Ethics Committee, and in accordance with the Declaration of Helsinki. Human blood was collected in 3.8% (v/v) citrate by venepuncture from aspirin free, healthy volunteers after obtaining their informed consent. Detailed method is available in the supplementary information. All methods performed in the study were carried out in accordance with the University of Reading guidelines and regulations concerning ethical approval, health and safety, the use of animals in experimentation and research quality assurance. Experimental protocols were approved by the University of Reading Research Ethics Committee and the Animal Welfare Ethical Review Board. Experiments using animals were approved and performed in accordance with a licence issued by the UK Home Office

### Platelet preparation, aggregation and dense granule secretion assays

Platelet aggregation and dense granule secretion in washed platelets was determined using lumiaggregometry by measuring changes in optical density and ATP release, respectively. Detailed method is available in the supplementary information.

### Immunofluorescence microscopy

Human (in PRP) stimulated with or without thromboxane A_2_ receptor agonist, U46619 (5 µM), were left to settle on poly-L-lysine coverslips for 1 hour at 37 °C before permeabilisation and blocking (0.2% Triton-X-100, 1% BSA, 2% donkey serum). Coverslips were then incubated with primary antibodies (PXR or RXR and GPIb) overnight (4 °C) and washed in PBS before staining with Alexa-fluor conjugated secondary antibodies for 1 hour at room temperature. Platelets were imaged on a Nikon A1-R confocal microscope (100X magnification oil immersion lens). Detailed method is available in the supplementary information.

### Immunoblotting and immunoprecipitation

Washed platelets (4 × 10^8^ or 8 × 10^8^ cells/ml) were lysed in an equal volume of NP40 buffer (300 mM NaCl, 20 mM Tris base, 2 mM EGTA, 2 mM EDTA, 1 mM PMSF, 10 µg/ml aprotinin, 10 µg/ml leupeptin, 0.7 µg/ml pepstatin A, 2 mM sodium orthovanadate, 2% NP-40, pH 7.3), and proteins of interest were isolated using 1 μg/mL of appropriate antibodies. Detailed method is available in the supplementary information. Immunoblotting was performed using standard techniques as described in the supplementary information. Levels of phosphorylated proteins were detected using fluorophore-conjugated secondary antibodies and visualised using a Typhoon FLA 9500 fluorimeter (GE healthcare) and quantified using Image Quant software version 8.1 (GE healthcare). Protein loading was assessed through reprobing for actin or 14-3-3ζ.

### Fibrinogen binding and alpha granule secretion

Activation of the integrin αIIbβ3 and alpha-granule secretion were measured by determining levels of fibrinogen binding and P-selectin exposure on the platelet surface by flow cytometry using FITC-conjugated anti-fibrinogen and PE/Cy5-labelled anti-CD62P antibody, respectively. Using a BD Accuri C6 flow cytometer, 10,000 events were analysed using the CFlow Sampler software. Detailed methods for both are available in the supplementary information.

### Calcium mobilization

PRP was loaded with Fura-2 AM (2 µM) for 1 h at 30 °C and then washed platelets were prepared. Fura-2AM-loaded platelets were incubated with PXR ligands or vehicle prior to their activation. The ratio of emission values (excitation at 340/380 nm) was recorded using a NOVOstar plate reader (BMG Labtech) and converted to calcium concentration. Detailed method is available in the supplementary information.

### Platelet spreading and clot retraction

Washed human platelets (2 × 10^7^ cells/ml) treated with or without PXR ligands were exposed to fibrinogen or collagen (100 µg/ml) coated coverslips for 45 minutes. Adhered platelets were fixed using 2% (v/v) paraformaldehyde and permeabilised with 0.2% (v/v) Triton-X-100. Thereafter, platelets were stained using Alexa488-conjugated phalloidin. Adherent platelets were imaged on a Nikon A1-R confocal microscope (100X magnification oil immersion lens). The number of platelets in different stages of spreading were obtained by counting, for each sample, the number of platelets in 5 randomly chosen fields of view. Clot retraction was studied in thrombin (1 U/mL) stimulated PRP treated with or without PXR ligands for a period of 1 hour (37 °C). Clot weight was measured as a marker of clot retraction. Detailed methods are available in the supplementary information.

### Thrombus formation *in vitro*

Human or mouse blood fluorescently labelled with lipophilic dye DIOC_6_ was pre-incubated with vehicle or PXR ligands and perfused over a collagen-coated (100 µg/ml) microfluidic biochip (Cellix) at an arterial shear rate (20 dyn/cm^2^). Images of thrombi were obtained using a using Nikon A1-R Confocal microscope (20X objective) and fluorescence intensity was calculated. Detailed method is available in the supplementary information.

### Thrombus formation *in vivo* and tail bleeding assay

Thrombosis in humanised PXR transgenic mice (hPXR) treated with SR12813 or vehicle that were administered intravenously was assayed following laser-induced injury by intravital microscopy. After laser-induced injury of the inner wall of cremaster muscle arterioles, accumulation of platelets was assessed. Fluorescence and bright-field images were recorded using an Olympus BX61W microscope with a 60 × /1.0 NA water immersion objective and a high-speed camera. Data was analysed using Slidebook5 software (Intelligent Imaging Innovations). Tail bleeding experiments following removal of the tail-tip were performed on 20–25 g hPXR mice, anesthetized with ketamine (100 mg/kg) and xylazine (10 mg/kg) injected intraperitoneally. Detailed methods are available in the supplementary information.

### Statistical analysis

Data were analysed using ANOVA with Bonferroni post-test as indicated, or where appropriate by t-test. The Mann-Whitney U test was used to analyse tail bleeding and thrombosis assay. Data represent mean ± SD and P < 0.05 was considered to be statistically significant. Statistical analysis was performed using GraphPad Prism 7.0 software (California, USA).

## Results

### PXR is present in human platelets

**Figure 1:**
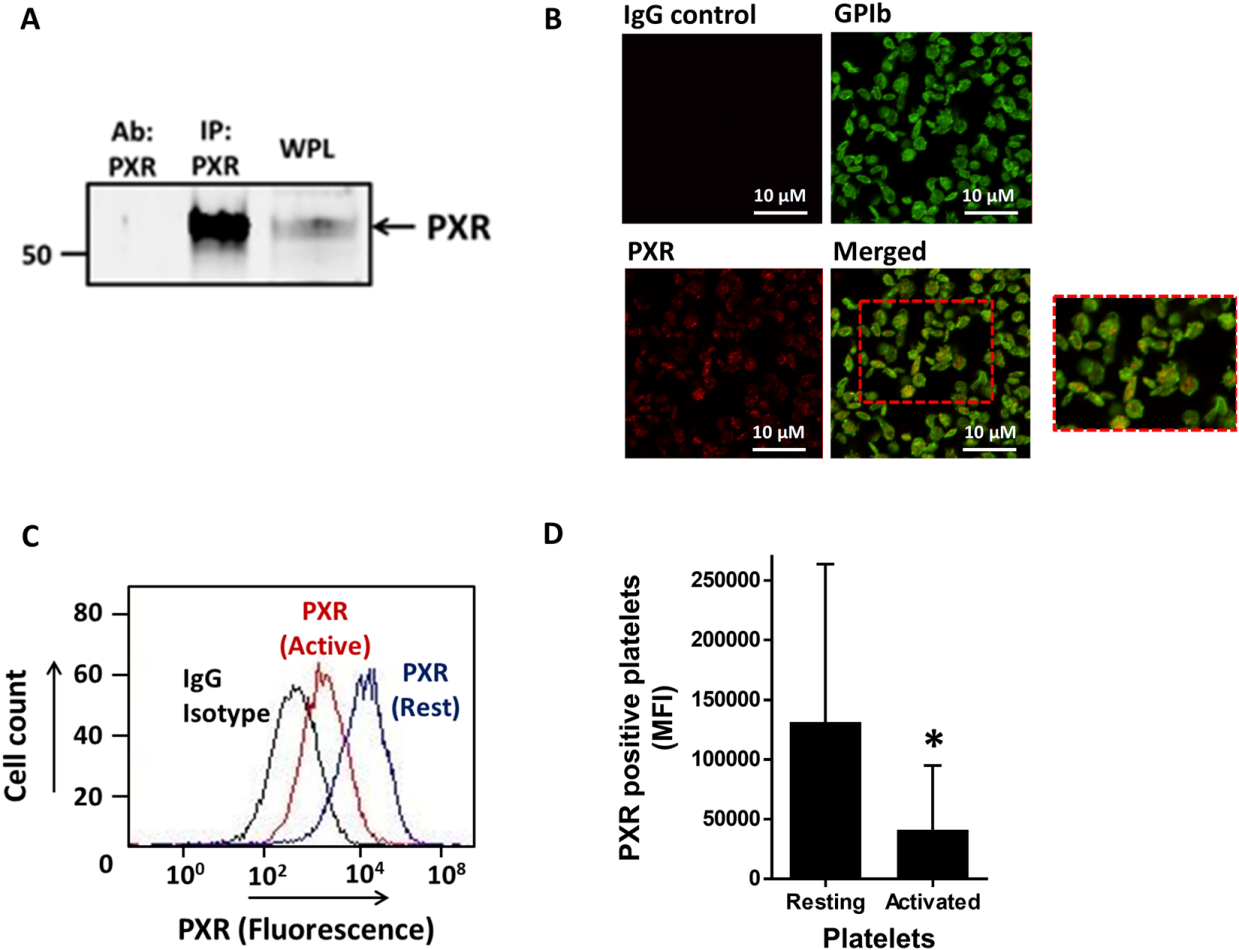
PXR is present in human platelets. (**A)** PXR was immunoprecipitated (IP: PXR) from human platelets (IP: PXR) using a mouse monoclonal antibody and blotted with a rabbit polyclonal antibody. Human whole platelet lysates (WPL) and antibody used to IP (Ab: PXR) PXR were used as positive and negative controls, respectively. Cropped western blot image is representative of 3 separate experiments using different donors. Full length blot is shown in Supplementary Fig. 8a **(B)** The localisation of PXR in human resting platelets was investigated using immunofluorescence microscopy. PXR (in red) and membrane GPIb receptors (in green) were stained using anti-PXR and anti-GPIb antibodies. Platelets without primary antibody treatment were used as negative controls. **(C)** Flow cytometry was used to examine the median fluorescence level of PXR in permeabilised resting and activated (with 0.1 U/ml thrombin) human platelets, incubated with a PXR antibody or the equivalent rabbit IgG isotype control. **(D)** Median fluorescence intensity (MFI) associated with the PXR positive platelets in resting and activated platelets. Data represent mean ± SD (N ≥ 3), *P < 0.05 was calculated by Student T-test. Figure adapted from corresponding PhD thesis^48^.

Initial analysis of platelet lysates for the presence of PXR revealed a weak band representing the receptor. Therefore, immunoprecipitation was performed to enrich the sample with PXR to enhance its detection. The protein was immunoprecipitated from platelet lysates using a mouse anti-PXR antibody (targeting amino acids 1–40) and its presence was confirmed by immunoblotting with a rabbit anti-PXR antibody (targeting amino acids 101–260) (Fig. 1A). The sub-cellular localisation of PXR was studied in resting human platelets using immunofluorescence microscopy. The PXR (red stain) was noted to be distributed inside the platelet cytosol (green stain marks platelet surface GPIb) in a punctate arrangement (Fig. 1B). Previously, it has been reported that NRs such as RXR and PPARγ are secreted from platelets upon their activation^19^. This was evaluated for PXR in resting and activated (0.1 U/ml thrombin) permeabilised platelets using flow cytometry. The level of fluorescence associated with PXR in thrombin-activated platelets was reduced, in comparison with resting platelets, suggesting a reduction in the number of PXR molecules present inside the activated platelets, consistent with release or secretion (Fig. 1C,D).

### PXR and RXR exists as heterodimers in human platelets

**Figure 2:**
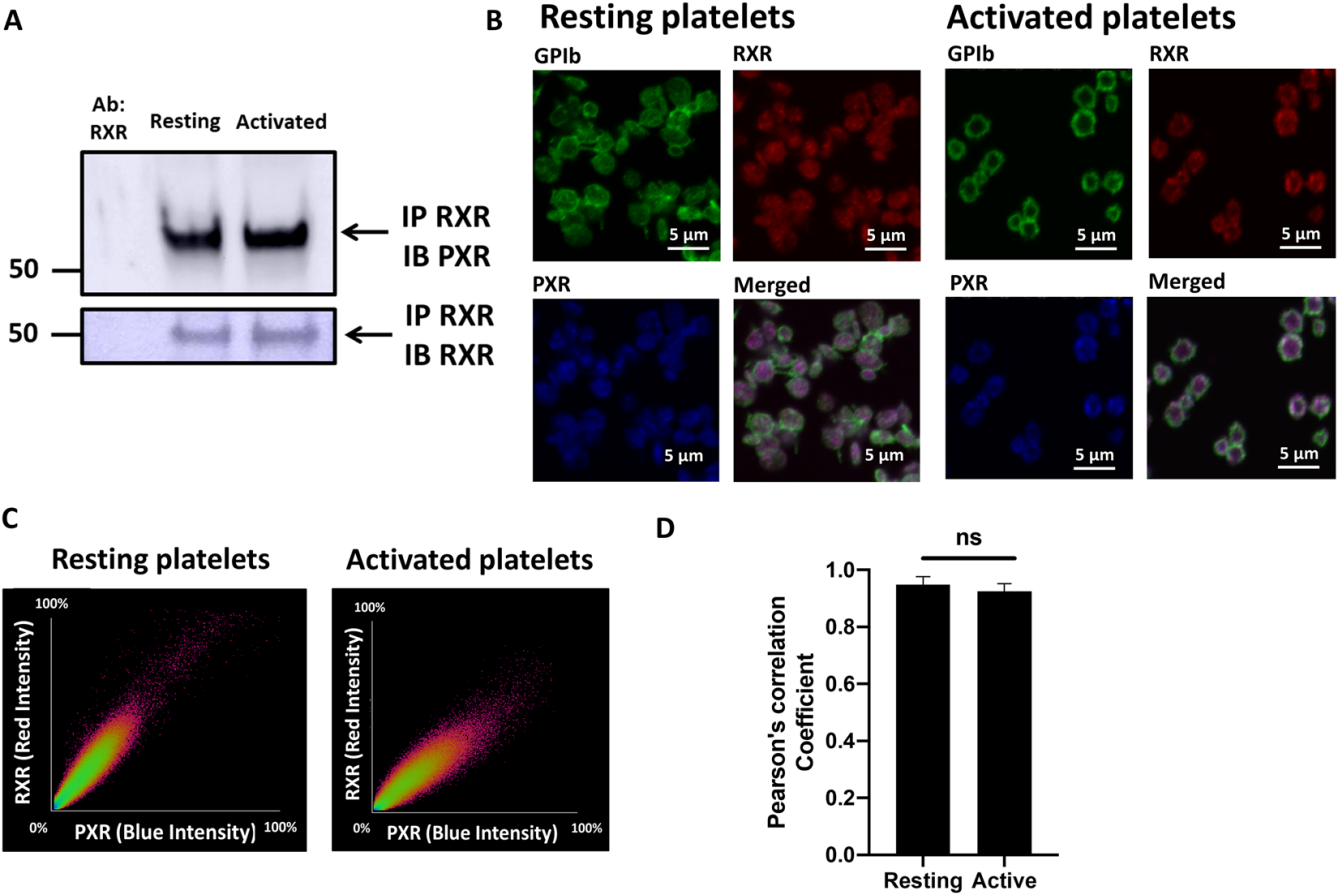
PXR and RXR interact and co-localise in human platelets. (**A**) RXR was immunoprecipitated from human washed platelets (8 × 10^8^ cells/ml) using a mouse monoclonal anti-RXR antibody. Immunoblot analysis was followed with the addition of a rabbit polyclonal anti-PXR antibody and its detection using a secondary antibody that does not recognize denatured IgG. The presence of RXR was also confirmed in the same samples. Cropped representative blot of 3 separate experiments is shown. Full length blot of PXR and RXR is shown in Supplementary Fig. 8Bi,ii respectively **(B)** Localisation of PXR and RXR in resting and activated (with 5 µM U46619 in the presence of integrelin) permeabilised human platelets was investigated using immunofluorescence microscopy. RXR (in red), PXR (in blue) and membrane GPIb receptors (in green) were stained using anti-RXR, anti-PXR and anti-GPIb antibodies respectively. Representative figures show the distribution of RXR and PXR in resting and activated platelets. **(C)** Scatter plots between the fluorescence intensity points of RXR and PXR in resting and activated platelets represent the degree of colocalisation between RXR and PXR. **(D)** The Pearson correlation coefficient (PCC) representing the degree of colocalisation between RXR-PXR in resting and activated platelets. PCC was quantified for 12 platelets using different fields. Data represent mean ± SD, **P < 0.01 and ***P < 0.001 was calculated by Student T-test. Figure adapted from corresponding PhD thesis^48^.

The formation of heterodimers between RXR and other NRs (PPARs, FXR, PXR, LXR) in nucleated cells is known^20^ and our previous work suggested the existence of RXR-LXR, RXR-PPARα and RXR-PPARγ heterodimers in human platelets^6^. The potential ability for PXR to interact with RXR in platelets was therefore examined. RXR and PXR were found to co-immunoprecipitate in both resting and activated platelets (Fig. 2A). Using immunofluorescence microscopy, a high degree of colocalisation between RXR (red) and PXR (blue) was observed in both resting and U46619-activated (5 µM) platelets (stained in green for GPIb (Fig. 2B)). A scatter plot demonstrated a clear relationship of fluorescence intensity points for RXR and PXR, clustering proportionally along a straight line (approximately 45 degrees to either axis), indicating a high level of colocalisation in resting and activated platelets (Fig. 2C). In alignment with this, the Pearson’s correlation coefficient (PCC), was found to be 0.94 and 0.92 between RXR and PXR in resting and activated platelets, respectively, indicating a strong co-localisation. There was no change in the extent of PXR-RXR colocalization between resting and activated platelets (Fig. 2D).

### PXR ligands inhibit platelet aggregation to a range of platelet activators

**Figure 3:**
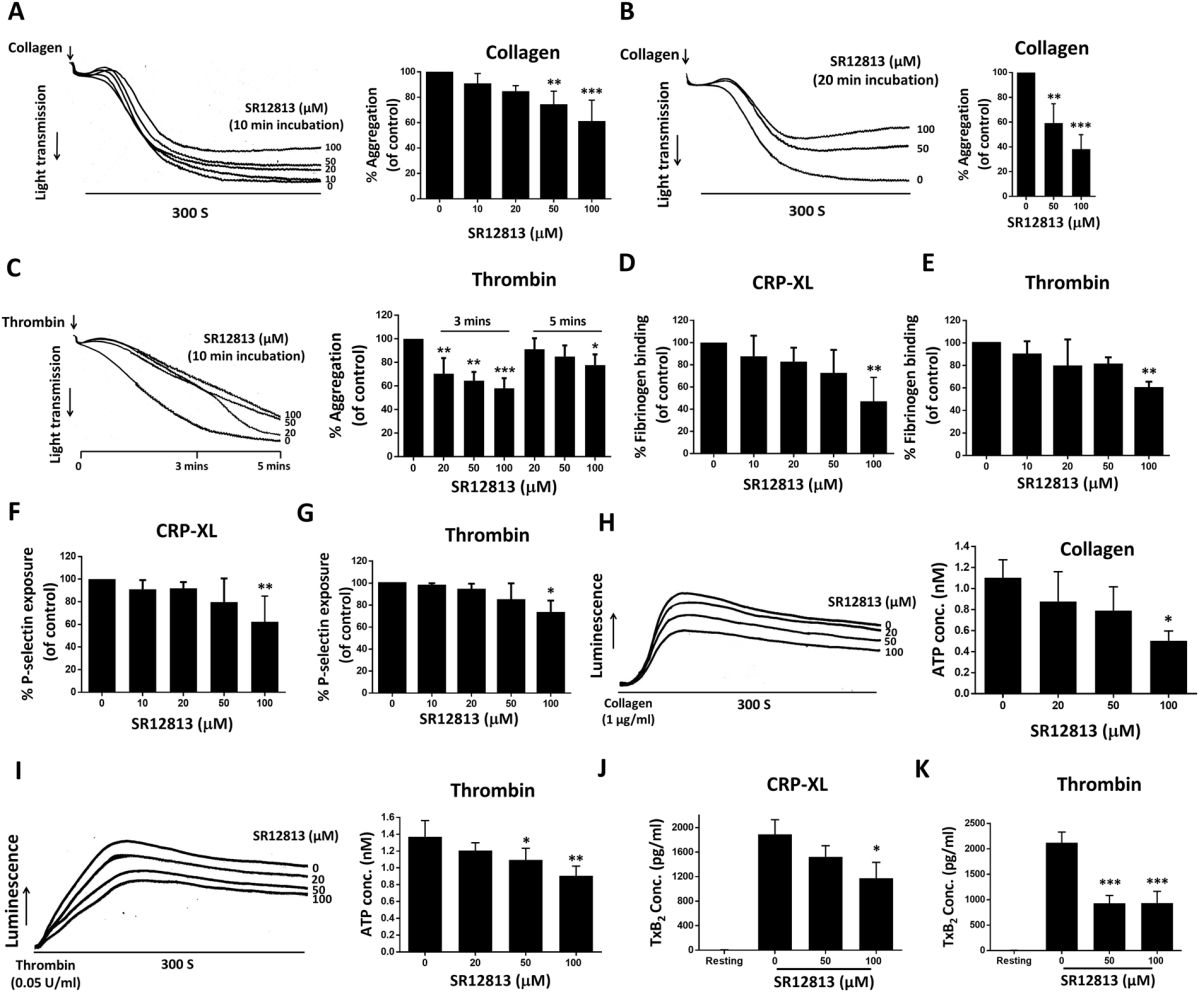
SR12813 inhibits platelet aggregation, integrin αIIbβ3 activation and secretion. Human washed platelets (4 × 10^8^ cells/ml) pre-treated with SR12813 or vehicle-control (DMSO, 0.1% v/v) were stimulated with **(A,B)** collagen (EC_50_: 0.5–0.8 µg/ml) or **(C)** thrombin (EC_50_: 0.03–0.04 U/ml). Representative aggregation traces are shown. Quantified data displays the percentage of aggregation (vehicle-treated samples represent 100% aggregation) at the end of 5 minutes. **(D)** Human PRP treated with SR12813 or vehicle for 10 minutes was stimulated with CRP-XL (EC_50_: 0.25 µg/ml) or thrombin (EC_50_: 0.05 U/ml) and fibrinogen binding to integrin αIIbβ3 was measured using flow cytometry. **(E)** P-selectin exposure was measured in SR12813 treated PRP, stimulated with CRP-XL (0.25 µg/ml) or thrombin (0.05 U/ml). Vehicle-treated control is defined as 100% fibrinogen binding and P-selectin exposure. ATP release was monitored for 5 minutes in washed platelets (4 × 10^8^ cells/ml), incubated with SR12813 or vehicle-control for 20 mins and stimulated with **(F)** collagen (1 µg/ml) or **(G)** thrombin (0.05 U/ml). Representative traces and quantified data are shown. Vehicle-treated samples represent 100% ATP secretion. **(H)** TxB_2_ production was evaluated in human washed platelets (4 × 10^8^ cells/ml) pre-incubated with SR12813 or vehicle control for 20 min and stimulated by CRP-XL (1 μg/ml) or thrombin (0.05 U/ml) for 5 minutes. Data represent mean ± SD (n ≥ 3), *P < 0.05, **P < 0.01 and ***P < 0.001 was calculated by one-way ANOVA. Figure adapted from corresponding PhD thesis^48^.

The effects of two structurally distinct PXR ligands, SR12813 and rifampicin^10^ were evaluated on platelet aggregation, stimulated by various platelet activators. To maintain consistency in results, the concentration of each platelet agonist for each donor was optimized to attain 50% maximal aggregation (EC_50_) in 5 minutes. Treatment of platelets with SR12813 for 10 minutes reduced platelet aggregation mediated by collagen (EC_50_: 0.5–0.8 μg/ml) by 27% and 40% at 50 and 100 μM SR12813 (Fig. 3A), respectively, compared to vehicle (DMSO 0.1% v/v). Similar levels of inhibition were observed with rifampicin treatment (Suppl. Fig. 1A). Increasing the incubation period of SR12813 (Fig. 3B) or rifampicin (Suppl. Fig. 1B) to 20 minutes strongly enhanced inhibition against collagen-stimulation, which may reflect the rate of transit of PXR ligands across the plasma membrane. Aggregation mediated by the G protein–coupled receptor (GPCR) agonist thrombin (EC_50_: 0.03–0.04 U/ml) was also attenuated by SR12813 or rifampicin (10-mins incubation). 50 and 100 μM of SR12813 demonstrated 35% and 42% reduction in aggregation respectively at 3-minute post-activation by thrombin in comparison with 5-minutes interval, which was around 15% and 23%, respectively (Fig. 3C). Similar observations were made with rifampicin (Suppl. Fig. 1C). In addition to this, both the PXR ligands inhibited U46619-mediated platelet aggregation (EC_50_ range: 0.2–0.4 μM) (Suppl. Fig. 1D,E) and ADP (EC_50_ range: 5–10 μM) (Suppl. Fig. 1F,G), indicating that the effects of PXR ligands are widespread (acting on both GPVI and GPCR-mediated platelet activation) and not restricted to a specific platelet activation pathway.

### PXR ligands inhibit integrin αIIbβ3 activation and platelet secretion

The affinity upregulation of integrin αIIbβ3 following platelet activation is essential for platelet aggregation. Therefore, the effects of PXR ligands on the extent of CRP-XL (EC_50_: 0.25 µg/ml) or thrombin (EC_50_: 0.05 U/ml) induced fibrinogen binding to integrin αIIbβ3 were evaluated in PRP using flow cytometry. In alignment with reduced aggregation, CRP-XL-induced fibrinogen binding was inhibited by 52% at 100 μM SR12813, in comparison to vehicle-control (DMSO 0.1% v/v) (Fig. 3D). Thrombin-stimulated fibrinogen binding was also attenuated by 100 µM SR12813 by 40% (Fig. 3E). Similar effects were demonstrated by rifampicin on CRP-XL or thrombin stimulation (Suppl. Fig. 2A). To investigate the effects of PXR ligands on degranulation, the extent of alpha and dense granules secretion was evaluated by measuring P-selectin exposure on the platelet surface and ATP release, respectively. SR12813 (Fig. 3F,G) and rifampicin (100 μM) (Suppl. Fig. 2B) caused 40% and 30% reduction in CRP-XL (0.25 µg/ml) or thrombin-stimulated (0.05 U/ml) P-selectin exposure, respectively, compared to vehicle (DMSO 0.1% v/v). ATP release following stimulation by either collagen (1 µg/ml) or thrombin (0.05 U/ml) was also attenuated following pre-treatment of platelets with SR12813 (Fig. 3H,I) or rifampicin (Suppl. Fig. 2C,D).

In addition to degranulation, activated platelets synthesise TxA_2_ from arachidonic acid through the actions of COX-1 and TxA_2_ synthase. TxA_2_ synthesis and release activate more platelets at the site of injury, thus, amplifying the aggregation response. SR12813 treatment significantly down-regulated both CRP-XL (1 μg/ml) or thrombin (0.05 U/ml) evoked TxB_2_ (a stable metabolite of TxA_2_) production by washed platelets (Fig. 3J,K). Higher concentrations of platelet agonists were used to ensure maximum secretion and synthesis of ATP and TxB_2_ from activated platelets for effective detection. Consequently, incubation time with PXR ligands was prolonged (20 mins) in these assays.

Collagen-evoked platelet aggregation depends partially on the release of secondary mediators. Since PXR ligands can inhibit aggregation instigated by both ADP and U46619, we investigated whether the inhibitory effects of PXR ligands against collagen-stimulation (10 µg/ml) are independent of their ability to reduce secondary mediator effects. Given the high concentration of collagen used in this assay, SR12813 (100 µM) on its own did not affect platelet aggregation (Suppl. Fig. 2E), however, the inhibitory effects of SR12813 (100 µM) were found to be additive to the inhibition caused by saturating concentrations of secondary mediator inhibitors; indomethacin (20 µM; TxA_2_ synthesis blocker) and ADP receptor antagonists (1 µM cangrelor and 100 µM MRS2179). This indicates that the effects of SR12813 on collagen-mediated platelet aggregation are not solely dependent on its effects on secondary mediator release during activation (Suppl. Fig. 2E).

### Species-specific effects and inhibition of thrombus formation *in vitro* by human and mouse PXR ligands

**Figure 4:**
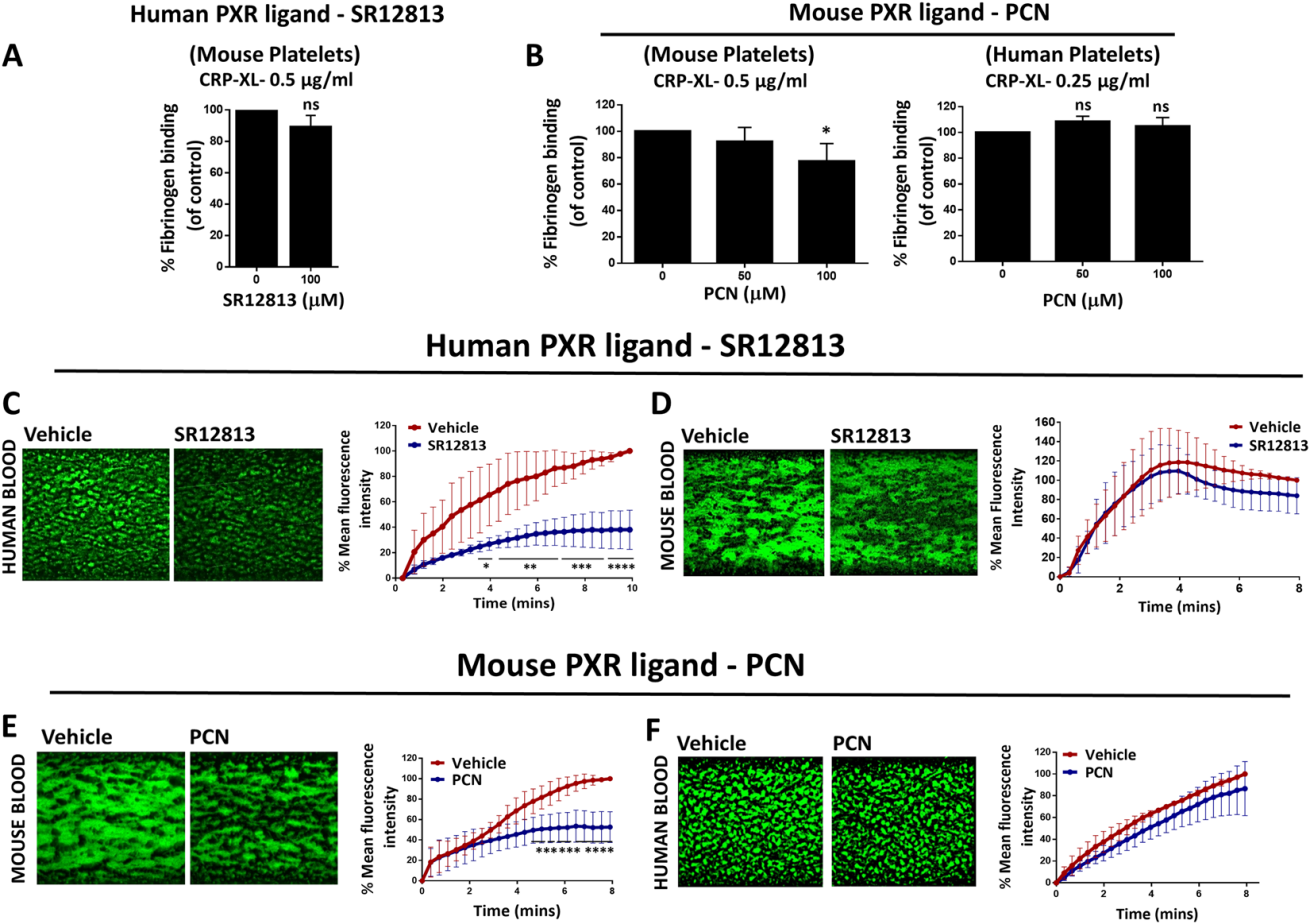
PXR ligands function in a species-specific manner and inhibit thrombus formation *in vitro*. Human or mouse PRP was treated with **(A)** SR12813 or **(B)** PCN or vehicle-control (0.1% DMSO v/v for SR12813 or 0.5% v/v DMSO for PCN) prior to stimulation with CRP-XL (0.25 µg/ml for human platelets and 0.5 µg/ml for mouse platelets). The level of fibrinogen binding to integrin αIIbβ3 was measured using flow cytometry. Human or mouse blood, incubated with DiOC6 (5 μM) were perfused through collagen-coated (100 μg/ml) microfluidic chips at arterial flow rate (20 dyne/cm^2^) after treatment with **(C,D)** SR12813 or **(E,F)** PCN or vehicle-control (0.1% DMSO v/v for SR12813 or 0.5% v/v DMSO for PCN) for 20 minutes. Representative images display thrombus formation. Quantified data represent mean thrombus fluorescence intensity normalised to fluorescence level of the vehicle-treated sample obtained at the end of the assay. Data represent mean ± SD (n ≥ 3) where *P < 0.05, **P < 0.01, ***P < 0.001 and ****P < 0.0001 was determined by student t-test or one-way ANOVA for fibrinogen binding assay and two-way ANOVA for *in vitro* thrombus formation assay. Figure adapted from corresponding PhD thesis^48^.

As mentioned earlier, a dissimilarity exists in the sequence of the PXR LBD between species, with only 76% amino acid sequence similarity in the LBD between human and mouse PXR^10^. This results in a high degree of inter-species differences in ligands that activate PXR. This property was explored to assess whether the effects of PXR ligands are likely to be mediated through binding to PXR protein in platelets. Consequently, the effects of human (SR12813) or mouse (PCN) PXR ligands on CRP-XL-stimulated fibrinogen binding in human and mouse platelets were investigated. It was not possible to directly compare the responses of human and mouse platelets as their activation profile is quite different towards the similar concentrations of platelet agonists. For instance, a concentration of 0.25 μg/ml of CRP-XL was sufficient to activate human platelet samples to study the effects of human PXR ligands, however, this produced a modest effect on mouse platelets. Consequently, the CRP-XL concentration was enhanced to 0.5 μg/ml for experiments using mouse PRP. In comparison to vehicle-control (DMSO 0.1% v/v), 100 μM of SR12813 reduced CRP-XL-stimulated (0.25 μg/ml) fibrinogen binding in human platelets by 50% (Fig. 3D). However, SR12813 did not cause any change in mouse platelet responses stimulated with CRP-XL (0.5 μg/ml) (Fig. 4A). Similarly, mouse PXR ligand, PCN (100 μM), inhibited CRP-XL-evoked fibrinogen binding in mouse platelets by 25% in comparison to vehicle-control (DMSO 0.5% v/v), whereas, no effect was observed in human platelets (Fig. 4B). These findings not only demonstrate species-specific effects of PXR ligand but also provides evidence that the effects of PXR ligands are mediated via PXR in human and mouse platelets.

Given the ability of PXR ligands to regulate numerous aspects of platelet activation, we investigated their effects on thrombus formation *in vitro*. Treatment with SR12813 (100 µM) for 20-minutes inhibited thrombus development in contrast to vehicle (DMSO 0.1% v/v) (Fig. 4C). Rifampicin (100 µM) treatment also reduced thrombus formation (Suppl. Fig. 3A). Furthermore, the initial kinetics of thrombus-development in PXR ligand-treated samples were inhibited in comparison to vehicle-control, which might be due to reduced adhesion of platelets to collagen.

The thrombus formation assay was also performed to study the species-specific effects of PXR ligands. In comparison to vehicle (DMSO 0.5% v/v), 20-minutes treatment with mouse PXR ligand, PCN (100 μM), caused a 50% reduction in thrombus formation in mouse blood (Fig. 4E). In contrast, thrombus formation in PCN (100 μM) treated human blood was not altered (Fig. 4F). To further confirm the species-specific nature of PXR ligands, the effect of human PXR ligand (SR12813) was evaluated in mouse blood. As discussed previously, SR12813 reduced thrombus formation in human blood (Fig. 4C), but thrombus development in mouse blood treated with SR12813 was similar to vehicle-treated samples (Fig. 4D).

### PXR ligands inhibit thrombosis and haemostasis in mice

Given the negative-regulation of thrombus formation *in vitro*, the acute effect of human PXR ligand (SR12813) was evaluated *in vivo*. As explained earlier, PXR ligands exhibit species-specific responses in platelets; therefore, the effects of SR12813 were studied on humanised PXR transgenic mice (hPXR). These mice lack the endogenous PXR gene, which is replaced with human PXR gene and is reported to be responsive towards human PXR ligands and not to mouse PXR ligand^21–24^. Prior to the investigation, the expression levels of integrin α2β1, αIIbβ3, GPIb and GPVI (Suppl. Figs. 3B–E) receptors on hPXR were compared with background C57BL/6 mice and were found to be similar.

**Figure 5:**
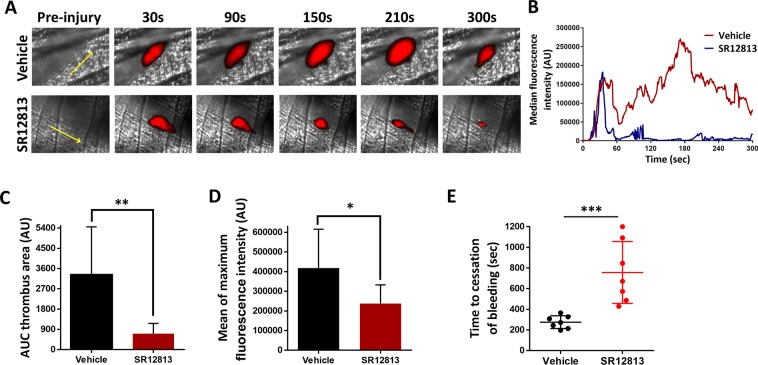
SR12813 inhibit thrombosis and haemostasis. Laser-induced injury model was used to measure thrombosis in hPXR mice through intravital microscopy. Vehicle-control (DMSO 0.1% v/v) or SR12813 (100 μM) was administered intravenously to mice and incubated for 20 minutes. Platelets were labelled with Alexa488–conjugated anti-GPIb antibody. **(A)** Representative images of thrombi obtained at different time intervals. **(B)** Data represent median fluorescence intensity, measured for 8 to 10 thrombi from 3 mice each of control and SR12813 treated groups. **(C)** Thrombus-size was calculated using area under the median fluorescence intensity curve of each thrombi. **(D)** Mean of maximum fluorescence intensity of the thrombus. **(E)** Tail bleeding was performed on hPXR mice pre-treated with vehicle or SR12813 (100 μM) for 20 min (n = 7). Data represent mean ± SD (n ≥ 3) where *P < 0.05, **P < 0.01 and ***P < 0.001 was determined by nonparametric Mann–Whitney U test. Figure adapted from corresponding PhD thesis^48^.

The acute effects of PXR ligands on *in vivo* thrombus formation were evaluated by studying laser-induced thrombosis in mouse cremaster muscle arterioles. As shown in Fig. 5A, the initial kinetics of thrombus formation in SR12813 (100 µM) treated mice was similar to vehicle (DMSO 0.1% v/v) (Fig. 5B), however, the overall size of thrombi was substantially reduced by 80% (Fig. 5C). Moreover, maximum fluorescence intensity of thrombus was reduced by 45% in SR12813 treated mice, consistent with the formation of smaller thrombi (Fig. 5D). Together these results suggest PXR ligands elicit anti-thrombotic effects. We cannot exclude the effects of other cell types on thrombosis, although the level of inhibition in the presence of SR12813 was comparable to its ability to attenuate thrombus formation *in vitro*, assays where vasculature or endothelial cells are not present. This suggests that PXR-mediated down-regulation of thrombus formation might be independent of the effects from other vascular cell types.

Given the observed anti-thrombotic properties of PXR ligands, the effect of SR12813 on haemostasis was measured by a tail-bleeding assay performed on hPXR mice. The mean time to cessation of bleeding was prolonged to approximately 500 seconds in mice treated with SR12813, in comparison vehicle-treated mice (275 seconds), demonstrating that acute PXR ligand treatment increases bleeding and impairs haemostasis (Fig. 5E). The tail-bleeding assay takes into account both platelet-function and and the coagulation processes^25–27^ and therefore, besides the effects of PXR ligands on platelet activation, the effects of PXR ligands on coagulation cannot be excluded.

### PXR ligands inhibit outside-in signaling

**Figure 6:**
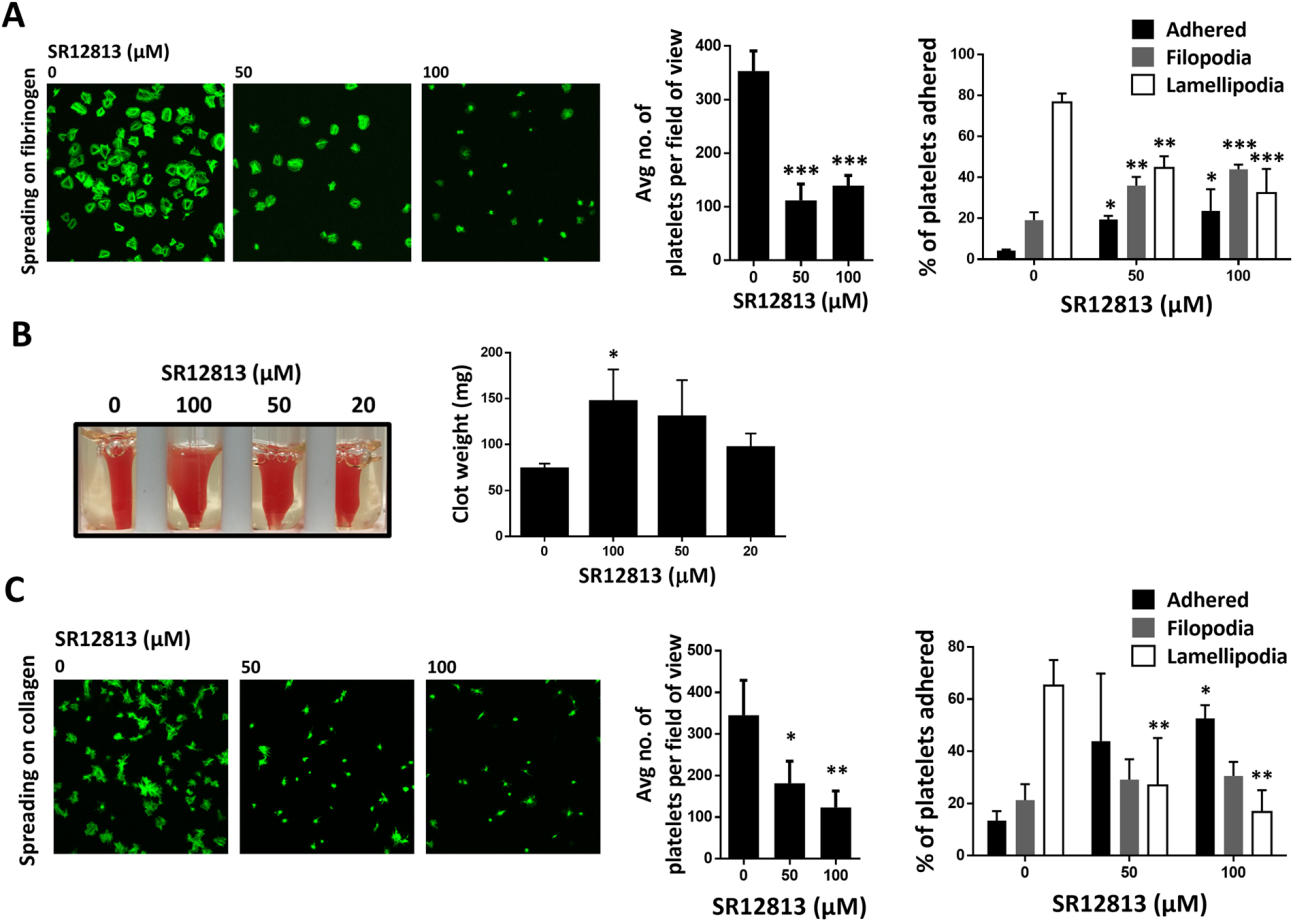
SR12813 inhibit outside-in signalling in platelets. Human washed platelets (2 × 10^7^ cells/ml) were treated with SR12813 (50 and 100 μM) or vehicle-control (DMSO 0.1% v/v) for 20 minutes and added onto **(A)** fibrinogen (100 μg/ml) or **(C)** collagen-coated coverslips for 45 mins. Platelets were stained using Alexa-Fluor 488 for visualisation using a Nikon A1-R confocal microscope (100X). 5 images were captured of each sample at random locations. Representative images of platelet adhesion and spreading are shown. Cumulative data of platelets adhered in each sample is shown. Spreading platelets were divided into 3 classes: (adhered but not spread; filopodia: spreading platelets and lamellipodia: fully spread). Results expressed (as relative frequency) as the percentage of the total number of platelets adhered. **(B)** Human PRP was incubated with SR12813 (20, 50 and 100 μM) or vehicle-control (DMSO 0.1% v/v) for 20 minutes. Extent of clot retraction was determined by comparing clot weight after 60 minutes. Representative image of clot retraction after the end of the assay is shown. Cumulative data represent clot weight (in mg) of samples treated with SR12813 compared with vehicle-control. Data represent mean ± SD (n ≥ 3), *P < 0.05, **P < 0.01 and ***P < 0.001 was calculated by one-way ANOVA. Figure adapted from corresponding PhD thesis^48^.

Binding of fibrinogen to integrin αIIbβ3 initiates outside-in signalling in platelets, which facilitates platelet spreading and clot retraction, required for the stability of the thrombus. Given the ability of PXR ligands to inhibit events associated with inside-out signalling such as aggregation, integrin αIIbβ3 upregulation and degranulation, their effects on outside-in signalling were also evaluated. In comparison to vehicle, fewer platelets were observed adhered to fibrinogen (in 45 minutes) following 20-minutes treatment with SR12813 (50 or 100 μM) (Fig. 6A). Additionally, SR12813 was also found to hinder platelet spreading with fewer platelets forming lamellipodia and increased numbers of cells remaining at the adhered or filopodial phases (Fig. 6A). Similar observations were noted with rifampicin treatment (Suppl. Fig. 4A). Consistent with this, an increase in clot weight (indicative of reduced clot retraction, a process driven by integrin αIIbβ3 outside-in signalling) was observed in samples treated with SR12813 (Fig. 6B) or rifampicin (Suppl. Fig. 4B) after 90 minutes, compared to vehicle-treated samples. These data along with previous findings suggest the ability of PXR ligands to modulate bi-directional signalling transmitted via integrin αIIbβ3.

To test whether the reduced adhesion and spreading were restricted to the functions of integrin αIIbβ3, similar experiments were performed on collagen (which is dependent on integrin α2β1 and GPVI). Platelet adhesion and spreading on collagen was also found to be inhibited with SR12813 (Fig. 6C) and rifampicin (Suppl. Fig. 4C) treatment. These findings along with observations of reduced thrombus formation (*in vitro*) suggest that PXR ligand-mediated reduction in thrombus formation might be partly due to the diminished platelet adhesion to collagen.

### PXR ligands inhibit GPVI-mediated signalling pathway

Having identified anti-thrombotic effects of PXR ligands, we determined the mechanism by which PXR ligands elicit anti-platelet activity. We and others have previously described the involvement of NRs in the regulation of platelet inhibitory signalling pathways, notably activation of the cGMP/PKG and cAMP/PKA linked pathways^1,4,6^. To determine whether PXR, which we have shown heterodimerises with RXR (which regulates PKA activity)^6^ acts in a similar manner, VASP S157 and VASP S239, markers of PKA and PKG activity respectively, were determined in PXR ligand treated platelets. Neither of the PXR ligands was found to activate PKA (Suppl. Fig. 5A,B) or PKG (Suppl. Fig. 5C,D) activity, indicating that PXR negatively regulates platelet function independently of these inhibitory signalling pathways.

**Figure 7:**
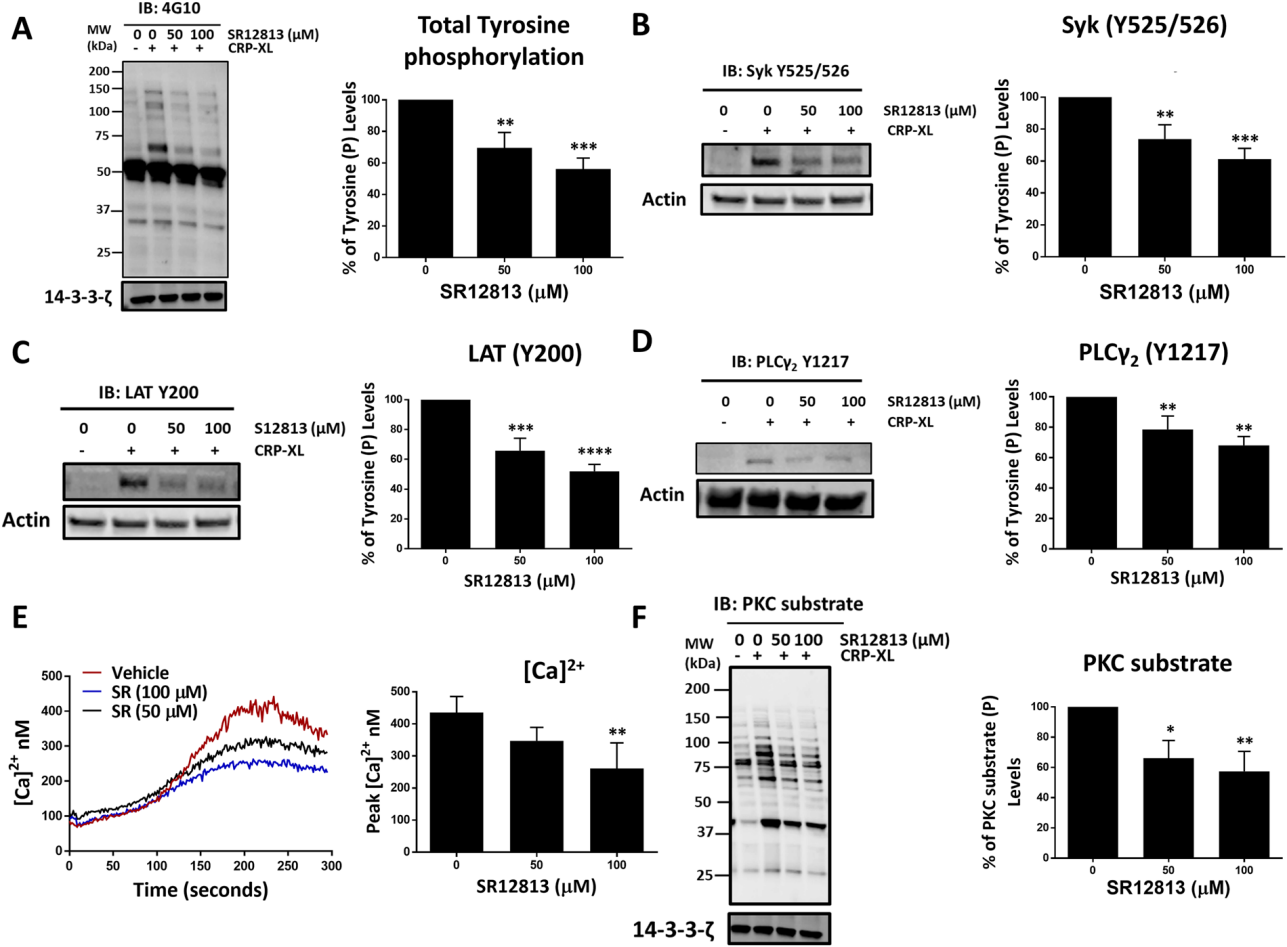
SR12813 negatively regulate GPVI-mediated signalling. Platelets (4 × 10^8^ cells/ml) were pre-treated with vehicle (DMSO 0.1% v/v) or SR12813 (0, 50 and 100 μM) for 20 minutes and stimulated with CRP-XL (1 μg/ml) for 90 seconds in the presence of indomethacin (20 μM), cangrelor (1 μM), MRS2179 (100 μM) and EGTA (1 mM). Samples were tested for **(A)** Total tyrosine, **(B)** Syk (Y525/526), (**C)** LAT (Y200), **(D)** PLCγ2 (Y1217) and **(F)** PKC substrate phosphorylation. Representative immunoblots are shown. Levels of phosphorylation were quantified and expressed as a percentage of untreated (vehicle) controls. 14–3–3-ζ or actin was used as a loading control. Full length blots are shown in supplementary Fig. 9 **(E)** Calcium mobilisation was evaluated in Fura-2AM loaded platelets (4 × 10^8^ cells/ml) incubated with SR12813 (50 and 100 μM) or vehicle-control (DMSO 0.1% v/v) for 20 min prior to stimulation with CRP-XL (0.25 μg/ml). Traces of CRP-XL-stimulated calcium mobilisation over a period of 5 minutes are shown. Cumulative data (peak calcium levels) of calcium mobilisation. Data represent mean ± SD (n ≥ 3) where *P < 0.05, **P < 0.01, ***P < 0.001 and ****P < 0.0001 was determined by One-way ANOVA. Figure adapted from corresponding PhD thesis^48^.

Since PXR ligands were observed to attenuate collagen/CRP-XL-mediated platelet activation, their effects on the GPVI-signalling pathway were investigated. Platelets were stimulated for 90 seconds under non-aggregating conditions [EGTA (1 mM), indomethacin (20 µM), cangrelor (1 µM) and MRS2179 (100 µM)] to block signalling via secondary mediators and ensure the study of primary GPVI-signalling. Consequently, CRP-XL concentration was increased (1 µg/ml) to visualise the phosphorylation of GPVI-signalling components by immunoblot analysis^6^. Consistent with our earlier observations, pre-treatment of platelets (20-minutes) with 50 and 100 µM of SR12813 (Fig. 7A) or rifampicin (Suppl. Fig. 6A) significantly reduced CRP-XL-evoked total tyrosine phosphorylation in comparison to vehicle (DMSO 0.1% v/v). Significant attenuation of early GPVI-signalling events, specifically phosphorylation of Syk (at its auto-phosphorylation site Y525/526) by SR12813 (Fig. 7B) or rifampicin (Suppl. Fig. 6B) was also observed. Following this, CRP-XL-stimulated phosphorylation of Linker for Activation of T cells (LAT) at site Y200 was also down-regulated by SR128123 (Fig. 7C) or rifampicin (Suppl. Fig. 6C) treatment along with the inhibition of PLCγ2 phosphorylation level (Fig. 7D) (Suppl. Fig. 6D) at Y1217.

As expected, PLCγ2-dependent downstream processes such as calcium-mobilisation were inhibited by both SR1813 (Fig. 7E) and rifampicin (Suppl. Fig. 6E) treatment, along with the reduction of protein kinase C (PKC) phosphorylation (Fig. 7F) (Suppl. Fig. 6F), both of which are essential for the regulation of cytoskeletal rearrangement, degranulation and integrin αIIbβ3 upregulation^28^. Together, these observations highlight a potential role for PXR ligands in regulating GPVI-receptor signalling.

### Inhibition of Src family kinases (SFKs) as a general mechanism for PXR function

**Figure 8:**
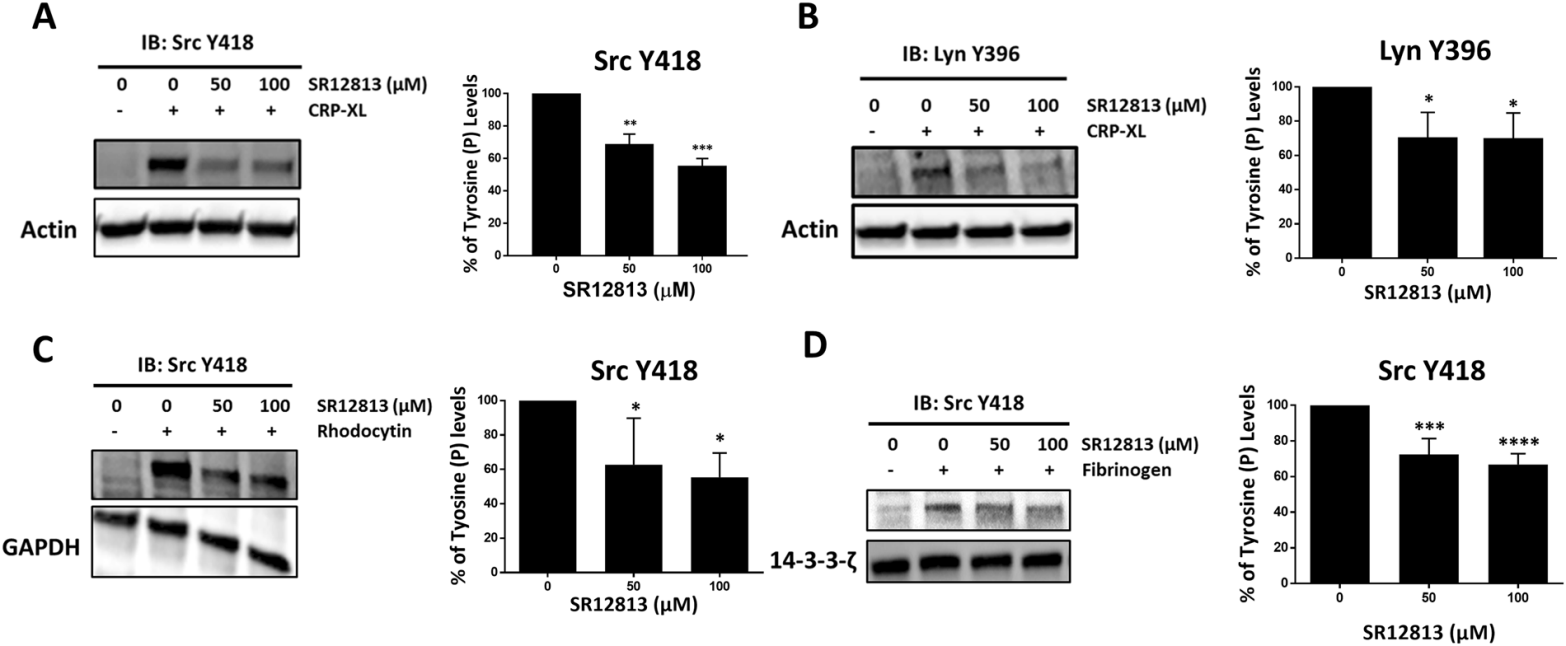
SR12813 inhibits tyrosine phosphorylation of SFKs proximal to GPVI, CLEC-2 and integrin αIIbβ3 receptors. Platelets (4 × 10^8^ cells/ml) were pre-treated with vehicle-control (DMSO 0.1% v/v) or SR12813 (0, 50 and 100 μM) for 20 minutes and stimulated for **(A, B)** 90 seconds with CRP-XL (1 μg/ml) or **(C)** 120 seconds with rhodocytin (100 nM) in the presence of indomethacin (20 μM), cangrelor (1 μM), MRS2179 (100 μM) and EGTA (1 mM). **(D)** Washed platelets (4 × 10^8^ cells/ml), pre-treated with SR12813 (0, 50 and 100 μM) or vehicle-control were exposed to fibrinogen-coated wells (100 μg/ml) of a tissue culture plate and allowed to adhere for 30 minutes. Samples were tested for Src (Y418) or Lyn (Y396) phosphorylation. Representative immunoblots are shown. The phosphorylation levels were quantified and expressed as a percentage of untreated (vehicle) controls. Actin was used as a loading control. Full length blots are shown in supplementary Fig. 9 and 10. Results are mean ± SD (n ≥ 3), *P < 0.05, **P < 0.01, ***P < 0.001 and ****P < 0.0001 was calculated by one-way ANOVA. Figure adapted from corresponding PhD thesis^48^.

Platelet function stimulated by GPVI, integrin αIIbβ3 and GPCRs are all dependent on signalling via SFKs^29^. The down-regulation of both early and late GPVI signalling events suggested the involvement of specific target elements of PXR ligands in GPVI signalling, facilitating this cascade of inhibition. Given the down-regulation of Syk phosphorylation, these elements are likely to act at a level that is upstream of Syk, such as SFKs. Therefore, the regulation of SFK activity by PXR ligands was monitored. Treatment with SR12813 (Fig. 8A) and rifampicin (Suppl. Fig. 7A) (50 and 100 µM) reduced CRP-XL-induced autophosphorylation of Src at Y418^30^ and Lyn at Y396^31^ (Fig. 8B) (Suppl. Fig. 7B), in comparison with vehicle (DMSO 0.1% v/v). To determine whether the regulation of SFKs marks a general mechanism of action for PXR ligands, signalling pathways initiated by other platelet receptors dependent on SFKs (such as CLEC-2 and integrin αIIbβ3) were examined^29^.

As was observed with other platelet agonists, SR12813 (Suppl. Fig. 7C) and rifampicin (Suppl. Fig. 7D) were found to attenuate platelet aggregation stimulated by CLEC-2 agonist rhodocytin (100 nM). Consistent with this, SR12813 (Fig. 8C) and rifampicin (Supplemental Fig. 7E) were also able to significantly diminish rhodocytin (100 nM) induced Src phosphorylation (Y418). The stimulation time with rhodocytin was enhanced (120 secs) to detect phosphorylation considering the long lag-phase associated with the initiation of rhodocytin-evoked platelet aggregation. To further support a general role for PXR ligand-dependent down-regulation of SFK activity, Src phosphorylation was also found to be attenuated in platelets treated with SR12813 (Fig. 8D), following adhesion to fibrinogen (100 µg/ml). Interestingly, no alteration in Src phosphorylation was observed in rifampicin-treated samples (Supplemental Fig. 7F). This might be attributed to the experimental challenges of examining signalling stimulated by fibrinogen under static conditions. Altogether these findings suggest that PXR ligands potentially mediate their inhibitory effects on platelet function via the down-regulation of SFKs activity.

## Discussion

Platelets are vital therapeutic targets for the treatment of cardiovascular diseases including atherothrombosis^32^. Current treatment includes the usage of anti-platelet drugs/therapies that prevent platelet activation by inhibiting different platelet signalling pathways^33^. These treatment regimens are effective, however, numerous side-effects (such as bleeding and drug resistance) limit their successful use^34^. Therefore, the development of newer strategies with minimal side-effects is required. NRs expressed in platelets have been proposed to exhibit antithrombotic effects^35^. In this study, we report the expression of PXR in platelets and the ability of its ligands to regulate platelet function and thrombosis.

Acute effects of PXR ligands were investigated, noting that the principal ligands for this receptor required high concentrations (10–100 µM for this study) to elicit acute biological effects^36^. It is reported that the ligands that activate PXR require micromolar concentrations, generally two to three orders of magnitude higher than concentrations found circulating in plasma^36^ and lower than the concentrations used in this study. Sub-micromolar concentrations of PXR ligands are sufficient to elicit genomic effects through regulation of gene expression in nucleated cells. Notably, the work presented here reveals the non-genomic effects of these ligands in anucleated platelets. Given the likely differences in the mechanism of genomic and non-genomic functions of NRs, including PXR, it is not possible to directly compare features of these responses such as EC_50_. Indeed, our group has previously shown that NRs (such as PPARγ, LXR, RXR, and FXR) in platelets regulate non-genomic functions that are distinct from genomic regulation^2,4–6,35^. Genomic effects of NRs are usually determined via cell-based reporter assays that involve exposing transcription factors (such as PXR) to their ligands for prolonged durations (hours or days)^10,37^, whereas the time frame in which non-genomic effects are elicited ranges from a few seconds to a few minutes and appears to require acute exposure to higher concentrations of NR ligands^38^. Further work will be required to establish the molecular basis of PXR and other NRs in platelets.

Treatment of platelets with PXR ligands resulted in attenuation of multiple aspects of platelet activation including aggregation, integrin αIIbβ3 activation and granule secretion. A trend of inhibition (although non-significant) was observed at low concentrations (10 and 20 μM) of PXR ligands. This is relevant as clinical administration of rifampicin (600 mg) to treat tuberculosis can achieve such peak plasma levels^39–41^. Increasing the acute treatment time of platelets with PXR ligands was found to enhance the anti-platelet effects of PXR ligands and as such further work is needed to determine the effects of chronic exposure to low concentrations of PXR ligands on platelet activity and thrombosis.

In platelets, apart from PXR, RXR also interacts with LXR^6^, PPARα^6^ and PPARγ^6,19^, although, the role of such heterodimers in non-genomic functions is unclear and requires further investigation. Platelets possess mRNA, capable of undergoing a minor level of translation^42^. Recently RARα was identified to regulate protein synthesis to some extent by its binding to a subset of mRNAs in human platelets^43^. Hence, it is possible that other NRs including PXR (in a bound or unbound state with RXR) may also contribute to some level of protein translation even in the absence of a nucleus^44,45^. To date, we have found no evidence to indicate the formation of NR homodimers in platelets.

PXR ligands down-regulated CRP-XL-mediated calcium-mobilisation and activation of PKC. This observation coupled with the previously reported abilities of PPARγ^2^, RXR^6^, LXR^5^ and FXR^4^ ligands to modulate calcium-mobilisation identifies a potentially common and fundamental role of NR ligands in regulating calcium homeostasis in platelets. In addition to PXR, other NRs such as LXR^5^ and PPARγ^2^ have also been identified to regulate the GPVI signalling pathway, whereas RXR^6^, PPARα^1^, PPARβ^46^ and FXR^4^ function by modulating cyclic nucleotide signalling in platelets. This suggests that platelet NRs have overlapping and distinct mechanisms of action in platelets^35^.

The inhibition of CRP-XL-induced downstream signalling events in platelets was identified to be an outcome of reduced phosphorylation of SFKs (Src and Lyn), key regulators of upstream signalling events. Besides GPVI, SFKs regulate signalling downstream of several other platelet receptors that include integrin αIIbβ3 and α2β1, CLEC-2, FcRγIIA and GPIb-IX-V receptor and therefore play a fundamental role in platelet activation^29^. SR12813 treatment negatively-regulated Src phosphorylation, proximal to integrin αIIbβ3, which ensures thrombus stability via the regulation of outside-in signalling. The mechanism by which, PXR regulates the phosphorylation of SFKs requires further exploration. Given the ability of NRs such as LXR^5^ and PPARγ^2^ to bind with key signalling molecules of the GPVI pathway and modulate signalling, it is possible that PXR follows a similar mechanism and interacts with one or more GPVI signalling components to regulate signalling. However, the interaction of PXR with SFKs, Syk, LAT or PLCγ downstream of GPVI was not observed in this study. There remains a range of other potential points of interaction to explore such as the GPVI/FcRγ complex that might facilitate this effect. Consistent with the inhibition of GPVI signalling, thrombus formation, both *in vitro* and *in vivo* was found to be reduced considerably by PXR ligands, which might be a combined outcome of reduced activity of SFKs downstream of GPVI, α2β1, GPIb-V-IX and integrin αIIbβ3. Furthermore, Src phosphorylation was also attenuated by PXR ligands, downstream of CLEC-2, which provides additional evidence that PXR ligands broadly affect the activity of SFKs in multiple signalling pathways and thus elicit their effects.

PXR is naturally promiscuous with its activating ligands representing a diverse array of structurally different compounds. However, this promiscuity is also specific in nature as its activators can structurally differ from non-activators by only a few atoms, suggesting that PXR binds to a diverse but precise array of compounds, a feature referred to as “directed promiscuity”^47^. In this study, we also devised strategies to rule out non-specific effects of PXR ligands. Firstly, we used two different PXR ligands (SR12813 and rifampicin) that have the highest affinity towards this receptor and are also structurally distinct from each other. In addition to this, PXR ligands demonstrated species-specific responses to PXR ligands. Human or mouse PXR ligands did not affect mouse or human platelet activation (as demonstrated via integrin αIIbβ3 activation and *in vitro* thrombus formation assays) respectively, while human PXR ligands significantly inhibited human platelets and mouse PXR ligands reduced mouse platelet activation. This is plausible only when the effects of PXR ligands are mediated via PXR. While systemic knock-out models are ideal to test the specificity of ligands, in many circumstances, deletion of transcription factors can lead to far-reaching effects on cell biogenesis and their functions, due to potential effects on the expression of many proteins. It is for this reason that humanised PXR mice were used in this study. In this study, the use of PXR antagonists was avoided, since most NR antagonists are characterised as such due to their effects on the regulation of gene expression. Since the effects of NR agonists on platelets are mediated by alternative, non-genomic mechanisms, and noting our previous observation that some NR antagonists (e.g. RXR) exert similar effects on platelets to respective receptor agonists^6^, we anticipated that this approach may produce a confusing picture. This reinforced our decision to explore the mode of action of PXR ligands in platelets using a genetic approach in mice.

In summary, our study demonstrates the anti-thrombotic properties of PXR ligands. In addition to the already identified anti-atherosclerotic effects of PXR^15–17^, these findings suggest the potential use of PXR as a cardio-protective drug target. PXR ligands were also associated with impaired haemostasis and therefore their potential development into effective therapeutic agents would require careful balancing of anti-platelet effects and bleeding risk.

## Supplementary information

Data 1
