# Supplementary material for: Non-genomic effects of the Pregnane X Receptor negatively regulate platelet functions, thrombosis and haemostasis
Source: Sci Rep. 2019 Nov 20;9:17210. doi: 10.1038/s41598-019-53218-x (PMC6868193; doi:10.1038/s41598-019-53218-x)
Supplement: Supplementary file 1 — Data 1 [file 41598_2019_53218_MOESM1_ESM.docx]

**Supplementary Information**

**Non-genomic effects of the Pregnane X Receptor negatively regulate platelet functions, thrombosis and haemostasis**

Gagan D. Flora^1, 2^ **^†^**, Khaled A. Sahli^1^ **^†^**, Parvathy Sasikumar^1,3^, Lisa-Marie Holbrook^1,4^, Alexander R. Stainer^1^, Sarah K. Alouda^1^, Marilena Crescente^1,5^, Tanya Sage^1^, Amanda J. Unsworth^1,6^ and Jonathan M. Gibbins^1^

^1^Institute for Cardiovascular and Metabolic Research, School of Biological Sciences, University of Reading, UK

^2^Department of Internal Medicine, University of Iowa, Iowa City, IA, USA

^3^Centre for Haematology, Imperial College London, London, UK

^4^School of Cardiovascular Medicine and Sciences, King’s College London, London, UK

^5^Centre for Immunobiology, Blizard Institute, Barts and The London School of Medicine and Dentistry, Queen Mary University of London, London, UK

^6^School of Healthcare Science, Manchester Metropolitan University, Manchester, UK

**Methods**

**Human platelet preparation**

Human blood was taken from consenting, drug-free volunteers on the day of the experiment according to the methodology approved by the University of Reading Research Ethics Committee. Blood was taken using 3.8% (w/v) sodium citrate and Acid Citrate Dextrose (ACD; 110 mmol/L glucose, 80 mmol/L citric acid, 120 mmol/L sodium citrate) as an anticoagulant. Whole blood was centrifuged at 102*g* for 20 minutes at 20°C to yield platelet-rich plasma (PRP). Where washed platelets were required, they were isolated from the PRP by further centrifugation at 1413*g* for 10 minutes at 20^o^C in the presence of 0.1 μg/ml prostacyclin to prevent activation. The supernatant was discarded in Klorsept disinfectant (Medentech, Wexford, Ireland) and the platelet pellet was resuspended in 25ml of modified Tyrodes-HEPES buffer (134 mmol/L NaCl, 0.34 mmol/L Na_2_HPO_4_, 2.9 mmol/L KCl, 12 mmol/L NaHCO_3_, 20 mmol/L HEPES, 5 mmol/L glucose, 1 mmol/L MgCl_2_, pH 7.3) and 3 ml of ACD in the presence of 0.1 μg/ml prostacyclin. Platelets were centrifuged at 1413*g* for 10 minutes at 20^o^C and resuspended to a density of 4x10^8^ cells/ml in modified Tyrodes-HEPES buffer using a platelet count obtained with a Z Series Coulter Counter (Beckman Coulter, CA, USA). Washed platelets were rested for at least 30 minutes at 30^o^C prior to the experiment to allow responses to recover. Platelet preparations typically contained fewer than 1 contaminating erythrocyte or leukocyte per 6500 platelets.

ADP-sensitive washed platelets were prepared by collecting blood into 3.8% (w/v) sodium citrate and centrifugation at 102*g* for 20 minutes at 20^o^C to yield PRP (without the addition of ACD). Platelets were isolated from the PRP by further centrifugation at 350*g* for 20 minutes. The supernatant was discarded, and the platelet pellet was re-suspended to a density of 4x10^8^ cells/ml in modified Tyrodes-HEPES buffer.

### Immunofluorescence microscopy

Human blood was collected in vacutainers containing sodium citrate as described previously. The blood was centrifuged at 100g for 20 minutes to collect PRP. Resting or activated platelets (stimulated with 5 µM U46619; in the presence of 4 µM integrillin) in PRP were fixed with an equal volume of 8% paraformaldehyde-PBS (PFA-PBS) to make a final concentration of 4% (v/v) and incubated for 15 min. Thereafter, platelets were centrifuged at 950g for 10 minutes. The supernatant was removed, and platelet pellet was resuspended in 2 ml of PBS-ACD (pH 6.1) for washing. Platelets were centrifuged for 10 minutes at 950g and resuspended in 1 ml of PBS-ACD to concentrate platelets. Platelets were centrifuged again at the same speed for 10 minutes and then resuspended in 500µl of 1% (w/v) BSA-PBS, to concentrate platelets even more. Poly-L-lysine coated-12mm coverslips (VWR micro cover glass No.1.5) were put in 6x6 culture plate and 90μl of platelets were added on each coverslip. Culture plates were placed at 37^o^C for 90 minutes. After 2-3 washes with PBS, samples were blocked with 0.2% (v/v) Triton-X-100, 2% (v/v) serum from same species as secondary antibody and 1% (w/v) protease-free BSA for 1h. Thereafter, anti-PXR and anti-GPIb primary antibodies diluted (1:100) in 0.2% (v/v) Triton-X-100, 2% (v/v) serum from the same species as secondary antibody and 1% (w/v) protease-free BSA were added and left overnight. The following day, samples were washed with PBS (2-3 times) and secondary antibodies (1:200) were added for 1 hour at room temperature. The unbound antibodies were washed off with PBS (2-3 times) and samples were fixed using 4% (v/v) PFA-PBS for 5 minutes. The coverslips were washed again with PBS (2-3 times). Coverslips were placed on glass slides after adding ProLong Gold Antifade mounting media (Life technologies). The slides were kept at room temperature until mounting media dried and then kept in the fridge until they were imaged using a Nikon A1-R confocal microscope (100x oil immersion).

### Platelet aggregometry

Light transmission aggregometry (LTA) was performed in an optical platelet aggregometer (Chrono-Log, PA, USA, and Helena Biosciences Europe, Gateshead, UK). Washed platelets (4x10^8^ cells/ml) were stimulated in the presence of agonist (collagen, CRP-XL, thrombin, U46619 or ADP) with continuous stirring (1200 rpm at 37^o^C) for 5 minutes and aggregation was measured as an increase in light transmittance. The effects of PXR ligands on platelet aggregation were measured by incubating washed platelets with PXR ligand dissolved in DMSO (final DMSO concentration in sample of 0.1% v/v) or vehicle control (containing, DMSO 0.1% v/v) for 10 or 20 minutes prior to the addition of agonist. The data was quantified by considering vehicle-treated samples as 100% aggregation and the level of aggregation obtained in PXR treated samples was normalised to it.

**Fibrinogen binding and alpha granule secretion**

Fibrinogen binding and P-Selectin exposure were measured using FITC-conjugated polyclonal rabbit anti-human fibrinogen antibody and PE/Cy5 mouse anti-human CD62P antibody, respectively, in a 96-well flat bottom plate. PRP was treated with PXR ligands or vehicle control for 10 minutes (containing, DMSO 0.1% v/v). 1 μl each of anti-fibrinogen and anti-CD62P antibody was added per 50 μl sample prior to stimulation with agonists (CRP-XL or thrombin) for 20 minutes with occasional gentle mixing. GPRP (25 μg/ml) was added in samples stimulated with thrombin to prevent fibrin polymerization. Reactions were stopped by adding 0.2% (w/v) formyl saline. Analyses were performed by flow cytometry using a BD Accuri C6 flow cytometer (BD Biosciences, Oxford, UK), and data were collected from 10,000 events [gated on platelets using FSC (forward scatter, limited between 1520-16000000) and SSC (side scatter, limited between 152-1600000)] and analysed using inbuilt BD Accuri C6 plus software, version 1.0.264.21.

**Measurement of PXR in platelets using flow cytometry**

To measure PXR within platelets, resting and activated (with 1 µg/ml CRP-XL in the presence of integrilin) human washed platelets (200 µl) at 4x10^8^ cells/mL were fixed by adding an equal volume 2% (w/v) formyl saline and permeabilised using 400 µl of BD Phosflow Perm Buffer III (BD Bioscience, Oxford, UK) for 1 h in ice. Platelets were then incubated with rabbit anti-PXR primary antibody (SantaCruz; sc-25381) for an hour. Following washing at 550g for 20 min, platelets were resuspended in HEPES buffer saline. Thereafter, platelets were incubated with an appropriate secondary Cy5-conjugated antibody (Invitrogen, Paisley UK) for an hour. Negative controls were set using an appropriate isotype control. Analyses were performed by flow cytometry using a BD Accuri C6 flow cytometer (BD Biosciences, Oxford, UK), and data were collected from 10,000 events [gated on platelets using FSC (forward scatter, limited between 1520-16000000) and SSC (side scatter, limited between 152-1600000)] and analysed using inbuilt BD Accuri C6 plus software, version 1.0.264.21.

### Dense granule secretion

Secretion of ATP from dense Granule upon platelet agonist stimulation was measured in washed platelets (4x10^8^ cells/ml) using a Lumi-aggregometer (model 700, Chronolo-Log, PA, USA) (Feinman et al., 1977). Washed platelets were added to a glass cuvette and incubated with the Chronolume reagent for 2 minutes, while stirring using the aggregometer. 2 nM ATP was added to this stirred suspension of platelets to set the ATP response baseline. The luminescence increase was observed using the AggroLink 8 software (Chrono-Log, PA, USA), with the luminescent gain adjusted until the ATP response was within the manufacturer-instructed range of 20-60%. These settings were saved and used for the rest of the experiment. Thereafter, washed platelets were incubated with PXR ligands or vehicle control (containing, DMSO 0.1% v/v) at 37°C for 20 minutes under non-stirring conditions. 2 minutes prior the end of incubation period, Chronolume reagent was added and stirred using the aggregometer. Washed platelets were then stimulated by the addition of agonist (collagen or thrombin) and the baseline was set. ATP release from dense granule was recorded for 5 minutes following the addition of agonist using the AggroLink 8 software, which calculates ATP secretion levels from the 2nM ATP standard.

###

### Measurement of intracellular calcium mobilisation

PRP was incubated with Dual excitation dye (at 340 and 380 nm), Fura-2AM (2 μM) for 1 hour at 30^o^C and was followed by centrifugation at 350g for 20 minutes. The platelet pellet was resuspended in modified Tyrodes-HEPES buffer (4x10^8^ cells/ml). Thereafter, Fura-2 AM loaded washed platelets were incubated with PXR ligands or vehicle control (containing, DMSO 0.1% v/v) for 10 minutes at 37^o^C prior to addition of platelet agonists (CRP-XL or thrombin). Fluorescence measurements (excitation 340 and 380 nm, emission 510 nm) were recorded for 5 minutes (1 measurement every 1.5s) using a NOVOstar plate reader. [Ca^2+^]_i_ was estimated by using the ratio of the 340 nm and 380 nm excited signals. Calibration was performed by treating an untreated sample with digitonin (50 μM) to lyse the platelets, which releases the Fura-2AM into the Tyrodes buffer, containing CaCl_2_ (2 mM), allowing measurement of the maximum fluorescence ratio. To calculate the minimum fluorescence ratio, Ca^2+^ ions were chelated by addition of 10 mM ethylene glycol-bis(β-aminoethyl ester)-N,N,N’,N’-tetraacetic acid (EGTA) and 10 mM TRIS base (added to ensure an alkaline pH for optimal Ca^2+^ buffering by EGTA). Auto-fluorescence was measured using unloaded platelets. Using these calibration values (maximum, minimum and autofluorescence), experimental [Ca^2+^]_i_ concentrations were calculated using the following equation:

$$[{Ca}^{2+}]_{i}= K_{d} \times\frac{S_{f}}{S_{b}} \times\frac{R-R_{min}}{R_{max}-R}$$

Where K_d_ is the dissociation constant of Fura-2AM (~224 nM). S_f_ and S_b_ are the values of the fluorescence at 380nm excitation (corrected to background auto-fluorescence), with zero or saturating [Ca]^2+^ respectively. R is the 340/380nm fluorescence ratio, corrected for background fluorescence. R_min_ and R_max_ are the ratio limits at zero or saturating [Ca]^2+^, respectively, adjusted using a viscosity constant of 0.85. This corrects for the effects of the cellular environment on the fluorescence of Fura-2.

###

### Platelet adhesion and spreading

Glass coverslips were placed in 6 well plates and coated with collagen or fibrinogen (100 μg/ml each) (in modified PBS) for 1 hour. 1% (w/v) BSA was then added onto coverslips and incubated for 1 hour to prevent platelets binding to the glass. The coverslips were washed 3 times with PBS. Washed platelets at a density of 2x10^7^ cells/ml were treated with PXR ligand or vehicle control (containing, DMSO 0.1% v/v) for 20 minutes, and then added onto coverslips and incubated for 45 minutes at 37^o^C. The supernatant was then removed from the coverslips, which were again washed 3 times with PBS. Platelets were then fixed with 0.2% (w/v) paraformaldehyde (PFA) for 10 minutes, the supernatant removed, and coverslips washed 3 times with PBS. Platelets were then permeabilised with 0.2% (v/v) Triton-X-100 for 5 minutes, and then the supernatant was removed and coverslips washed 3 times again with PBS. Alexa-Fluor 488 phalloidin was then added onto the coverslips for 1 hour, incubated in the dark, to label platelet F (filamentous) actin. The supernatant was removed, and coverslips washed 3 times with PBS. Coverslips were then mounted onto slides with the addition of Prolong Gold Antifade mounting media to preserve fluorescence. Samples were imaged, using a 100X oil immersion lens on a Nikon A1-R confocal microscope (Nikon, Tokyo, Japan). Fluorescence was excited at 488 nm with an argon laser and emitted at 500-520 nm, with images captured in one focal plane. Platelet adhesion data were obtained by counting the number of platelets on 5 images of each coverslip that were captured randomly. Platelets were scored as adhered (not spread), spreading (defined as extending filopodia) or spread fully (lamellipodia formed), and the relative frequency of each population was determined using ImageJ software.

### Clot retraction

The PRP was obtained as described earlier and was incubated with 2 µl of PXR ligands or vehicle control (containing, DMSO 0.1% v/v) for 20 minutes. Modified Tyrodes-HEPES buffer was added to test tubes, along with red blood cells, to allow visualization of the clot. This was followed by the addition of PRP treated with PXR ligand or vehicle control. Clot formation was initiated by adding thrombin (final concentration 1 U/ml) to the test tubes. A glass pipette was added to the centre of each test tube, around which the clot would form, and samples were placed in an incubator chamber at 37^o^C. Photographs were taken every 10 minutes and the assay was terminated after 60 minutes at which time the clot in the vehicle-treated samples were seen to have retracted completely. Clot weight was measured as a marker for clot retraction. Clots were removed from the glass pipettes and transferred into the pre-weighed microfuge tubes. Clot mass was determined by subtracting the weight of pre-weighed microfuge tubes from the weight of microfuge tubes containing clot.

### Western blotting

Human washed platelets were prepared at a density of 8x10^8^ cells/ml as described earlier and lysed by adding 6X Laemmli sample reducing buffer [4% (w/v) SDS, 20% (v/v) glycerol, 0.5M Tris, 0.001% (w/v) Brilliant Blue R and 10% (v/v) 2-mercaptoethanol]. Samples were heated to 95^o^C for 5 minutes before storing at -20^o^C until use.

To study cell signalling, human washed platelets were prepared at a density of 4x10^8^ cells/ml under non-aggregation conditions [indomethacin (20 µM), cangrelor (1 µM), MRS2179 (100 µM) and EGTA (1 mM). These platelets were treated with PXR ligands or vehicle control (containing DMSO, 0.1% v/v) for 20 minutes and then stimulated with platelet agonists in the aggregometer. Unstimulated or stimulated samples were lysed with 6X Laemmli sample reducing buffer and heated to 95^o^C for 5 minutes before storing at -20^o^C until use.

Proteins were separated by SDS-PAGE as described previously by Laemmli (1970), using 10% or 4-20% Mini-PROTEAN TGX precast protein gels. Samples were heated to 95^o^C for 5 minutes again prior to loading into gels, which were submerged in 1X Tris/Glycine/SDS buffer (25 mM Tris, 192 mM glycine, 0.1% SDS, pH 8.3) within a Mini-PROTEAN tetra vertical electrophoresis cell (Bio-Rad, CA, USA). Electrophoresis was run for 45 minutes or 1 hour at a constant voltage of 150V.

The separated proteins on gels were transferred to a polyvinylidene difluoride (PVDF) membrane using semi-dry western blotting (Trans-Blot SD Semi-Dry Transfer Cell; BioRad, CA, USA). A single piece of PVDF membrane soaked in methanol was placed below the resolving gel in the transfer cell. This arrangement of gel and PVDF membrane was sandwiched between 4 sheets of 3MM filter paper soaked in cathode buffer (25 mM Tris-base, 40 mM 6-amino-N-hexanoic acid; pH 9.4) placed at the top and 4 sheets of 3MM filter paper soaked in anode buffer (300 mM Tris-base, 20% (v/v) methanol; pH 10.4) placed at the bottom. A constant voltage of 15V was applied to this setup for 2 hours to facilitate efficient transfer of proteins from gel to membrane.

PVDF membranes were then transferred into a 5% (w/v) solution of bovine serum albumin (BSA) dissolved in Tris-buffered saline with Tween 20 (TBS-T) (20 mM Tris, 140 mM NaCl, 0.1% Tween, pH 7.6) to block the membrane for 1 hour at room temperature. Primary antibodies were added into a 2% (w/v) solution of BSA (dissolved in TBS-T) and membranes were incubated with these solutions overnight at 4^o^C on a rotator. Primary antibody solutions were removed from the PVDF membranes the next day and membranes were washed three times for 10 minutes each with TBS-T. Secondary antibodies were added to a 2% (w/v) BSA (dissolved in TBS-T) solution, which was then added to PVDF membranes and incubated in the dark at room temperature for 1 hour. PVDF membranes were washed three times again for 5 minutes each with TBS-T. PVDF membranes were scanned using a Typhoon FLA 9500 (Amersham Biosciences, Buckinghamshire, UK), and quantification of the fluorescence intensity of individual bands was determined using Image Quant software version 8.1 (GE healthcare).

###

### Immunoprecipitation

Washed human platelets were prepared (8x10^8^ cells/ml) as described previously. Cells were lysed on ice using an equal volume of 2X NP40 buffer (300 mM NaCl, 20 mM Tris, 10 mM EDTA, 2% v/v NP40; pH=7.3) containing protease inhibitors [Leupeptin (10 µg/ml), aprotinin (10 µg/ml), phenylmethylsulphonyl fluoride (1 mM) sodium orthovanadate (1mM) and pepstatin-A (25 µg/ml)]. The lysed platelets in NP40 buffer (1X) were incubated with primary antibody and Protein A/G magnetic beads (20 μl per 500 µl of lysate) at 4°C overnight. The following day, the beads were collected in Eppendorf tube using a magnetic stand and washed twice with NP40 buffer (1X) containing protease inhibitors and once with TBST. Thereafter, 100 μl of 2X Laemmli sample reducing buffer was added to the beads. The samples were then heated to 95°C for 5 minutes and kept at -20°C for use in Western blotting.

### *In vitro* thrombus formation under flow

Human or mouse whole blood was incubated at 30^o^C with 5 μM of the lipophilic dye DiOC6 for 1 hour. Microfluidic channels (Cellix, Dublin, Ireland) were coated with type I collagen (100 μg/ml) for one hour and excess collagen was washed with modified Tyrodes-HEPES buffer. Whole blood was incubated with PXR ligands or vehicle control (containing, DMSO 0.1% v/v DMSO) for 20 minutes prior to perfusion through the collagen-coated microfluidic channels at an arteriolar shear stress of 20 Dyne/cm^2^ (shear rate: 500 s^-1^). Fluorescence was excited at 488 nm with an argon laser and emission detected at 500-520 nm. The thrombus formation on the microfluidic chip was observed using a Nikon A1-R confocal microscope with a 20X objective and images (focused on a single section) were captured every 1 second for 600 seconds. Mean thrombus fluorescence intensity was calculated using NIS Elements software (Nikon, Tokyo, Japan).

### *In vivo* thrombus formation

The mice were anaesthetised by intraperitoneal injection of ketamine (125 mg/kg), xylazine (12.5 mg/kg) and atropine (0.25 mg/kg). Anaesthesia was maintained with 5 mg/kg pentobarbital as and when required. The cremaster muscle was exteriorized and the connective tissue removed, after which an incision was made, allowing the cremaster muscle to be affixed over a glass slide as a single sheet; the muscle preparation was hydrated throughout with buffer (135mM NaCl, 4.7mM KCl, 2.7mM CaCl_2_, 18mM NaHCO3, pH 7.4). SR12813, vehicle control (containing, DMSO 0.1% v/v) and DyLight 649 anti-GPIbα antibody (0.2 μg/g mouse weight; for platelet labelling) was infused into the mouse circulation through carotid artery cannula prior to the injury (performed using a Micropoint Ablation Laser Unit; Andor Technology PLC, Belfast, Northern Ireland). Thrombus formation was visualised after 20 minutes of the infusion of SR12813 or vehicle control using an Olympus BX61W1 microscope (Olympus Corporation, Tokyo, Japan). The images were captured both prior to and after the injury, using a Hamamatsu digital camera C9300 (Hamamatsu Photonics UK Ltd, Hertfordshire UK) charge-coupled device (CCD) camera in 640 x 480 format. Images were analysed using Slidebook 6 software (Intelligent Imaging Innovations, CO, USA). Following the procedure, mice were sacrificed in accordance with Home office licences and approval from the University of Reading local ethics review panel and Animal welfare and Ethics Research Board.

### Tail bleeding assay

On the day of the experiment, C57/BL6 or hPXR mice were anesthetised by intraperitoneal injection of ketamine (125 mg/kg) and xylazine (12.5 mg/kg) and SR12813 or vehicle control (containing, DMSO 0.1% v/v) was injected via the femoral vein. 20 minutes later, the tail tip was removed with a scalpel and the tail was immediately placed into tubes containing saline, in a manner that prevented the cut end of the tail from contacting the side of the tube. The time of bleeding was recorded until the blood flow had ceased. Following the procedure, or after 20 minutes, mice were sacrificed in accordance with Home office licences and approval from the University of Reading local ethics review panel and Animal welfare and Ethics Research Board.

**Supplementary figures**

**Suppl. Figure 1**


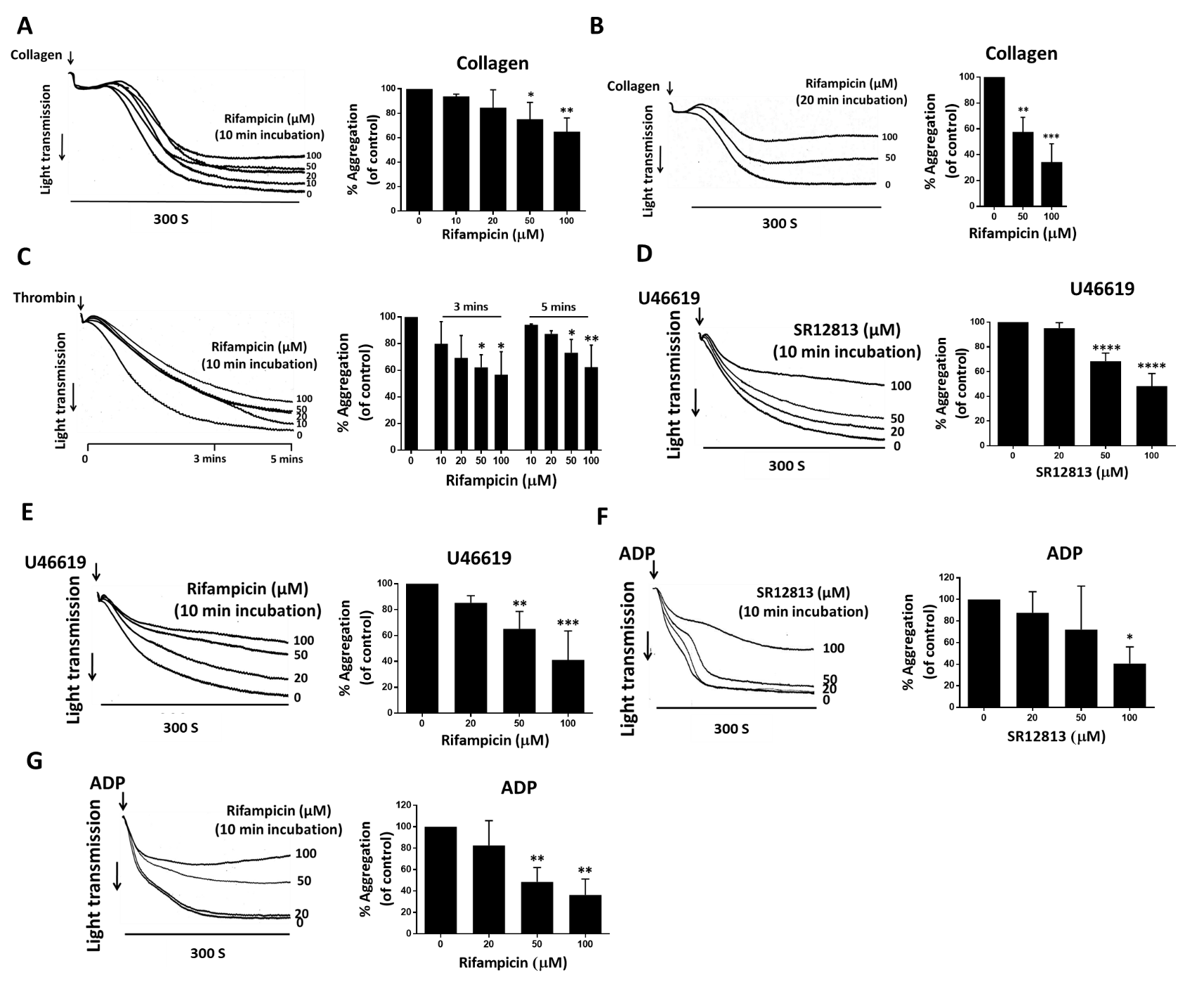


**Suppl. figure 1. PXR ligands inhibit platelet aggregation to a range of agonists.** Human washed platelets (4x10^8^ cells/ml) pre-treated with SR12813 or rifampicin or vehicle-control (DMSO, 0.1% v/v) were stimulated with **(A, B)** collagen (EC_50_: 0.5-0.8 µg/ml) or **(C)** thrombin (EC_50_: 0.03-0.04 U/ml) or **(D, E)** U46619 (EC_50_: 0.25 µM) or **(F, G)** ADP (EC_50_: 5-10 µM). Aggregation was measured for 300 seconds. Representative aggregation traces are shown. Quantified data displays the percentage of aggregation of SR12813 or rifampicin treated samples (vehicle-treated samples represent 100% aggregation) at the end of 5 minutes. Data represent mean±SD (n≥3), *P˂0.05, **P˂0.01, ***P˂0.001 and ****P˂0.0001 was calculated by one-way ANOVA. Figure adapted from corresponding PhD thesis - Non-genomic effects of the Pregnane X Receptor (PXR) and Retinoid X Receptor (RXR) in platelets^49^.

**Suppl. Figure 2**

**
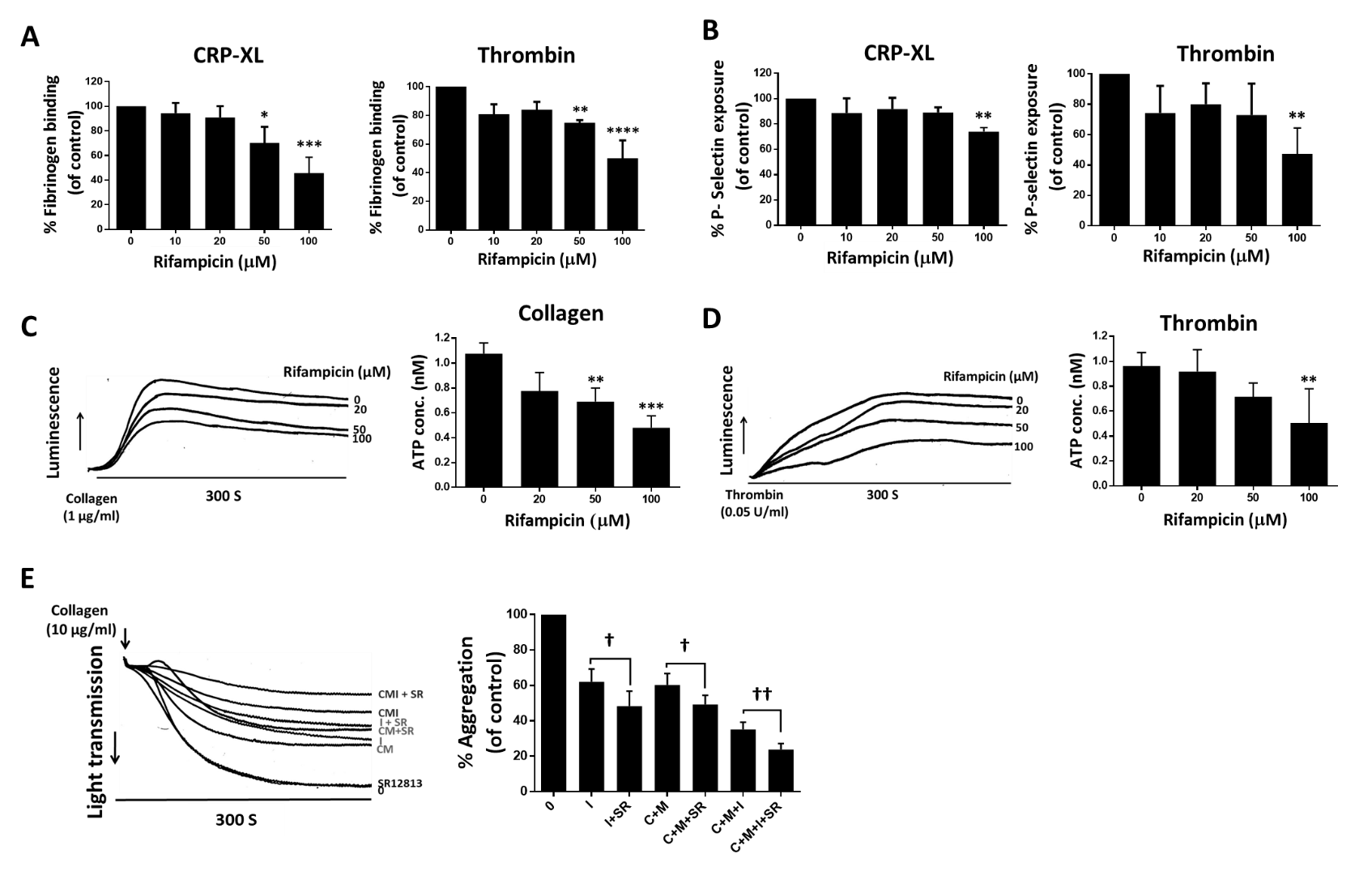
**

**Suppl. figure 2. Rifampicin attenuate fibrinogen binding to integrin αIIbβ3 and degranulation. (A)** Human PRP was treated with rifampicin or vehicle-control (DMSO 0.1% v/v) for 10 minutes prior to stimulation with CRP-XL (EC_50_: 0.25 µg/ml) or thrombin (EC_50_: 0.05 U/ml) and fibrinogen binding to integrin αIIbβ3 was measured using flow cytometry. **(B)** P-selectin was measured in rifampicin treated PRP and stimulated with CRP-XL (0.25 µg/ml) or thrombin (0.05 U/ml). Vehicle-treated control is defined as 100% fibrinogen binding and P-selectin exposure. **(C, D)** Changes in ATP release were monitored for 5 minutes in washed platelets (4x10^8^ cells/ml) incubated with rifampicin or vehicle-control for 20 mins and stimulated with collagen (1 µg/ml) or thrombin (0.05 U/ml). Representative traces and quantified data are shown. Vehicle-treated samples represent 100% ATP secretion. **(E)** Washed platelets (4x10^8^ cells/ml) pre-treated with saturating concentrations of indomethacin (20 μM) or ADP receptor antagonists - cangrelor (1 μM) and MRS2179 (100 μM) were stimulated with collagen (10 μg/ml) in the presence or absence of SR12813 (100 µM). Representative aggregation trace recorded for 5 minutes is shown. Quantified data for collagen-stimulated platelet aggregation in the presence or absence of SR12813, along with indomethacin (I+SR) or cangrelor and MRS2179 (C+M+SR) or all of them together (C+M+I+SR). ‘O’ signifies the sample stimulated with collagen in the absence of SR12813 and secondary mediator signalling blockers. Data represent mean±SD (n≥3), **^†^**P˂0.05 and **^††^**P˂0.01 was calculated by student t-test. **P˂0.01, ***P˂0.001 and ****P˂0.0001 was calculated by one-way ANOVA. Figure adapted from corresponding PhD thesis - Non-genomic effects of the Pregnane X Receptor (PXR) and Retinoid X Receptor (RXR) in platelets^49^.

**Suppl. Figure 3**

**
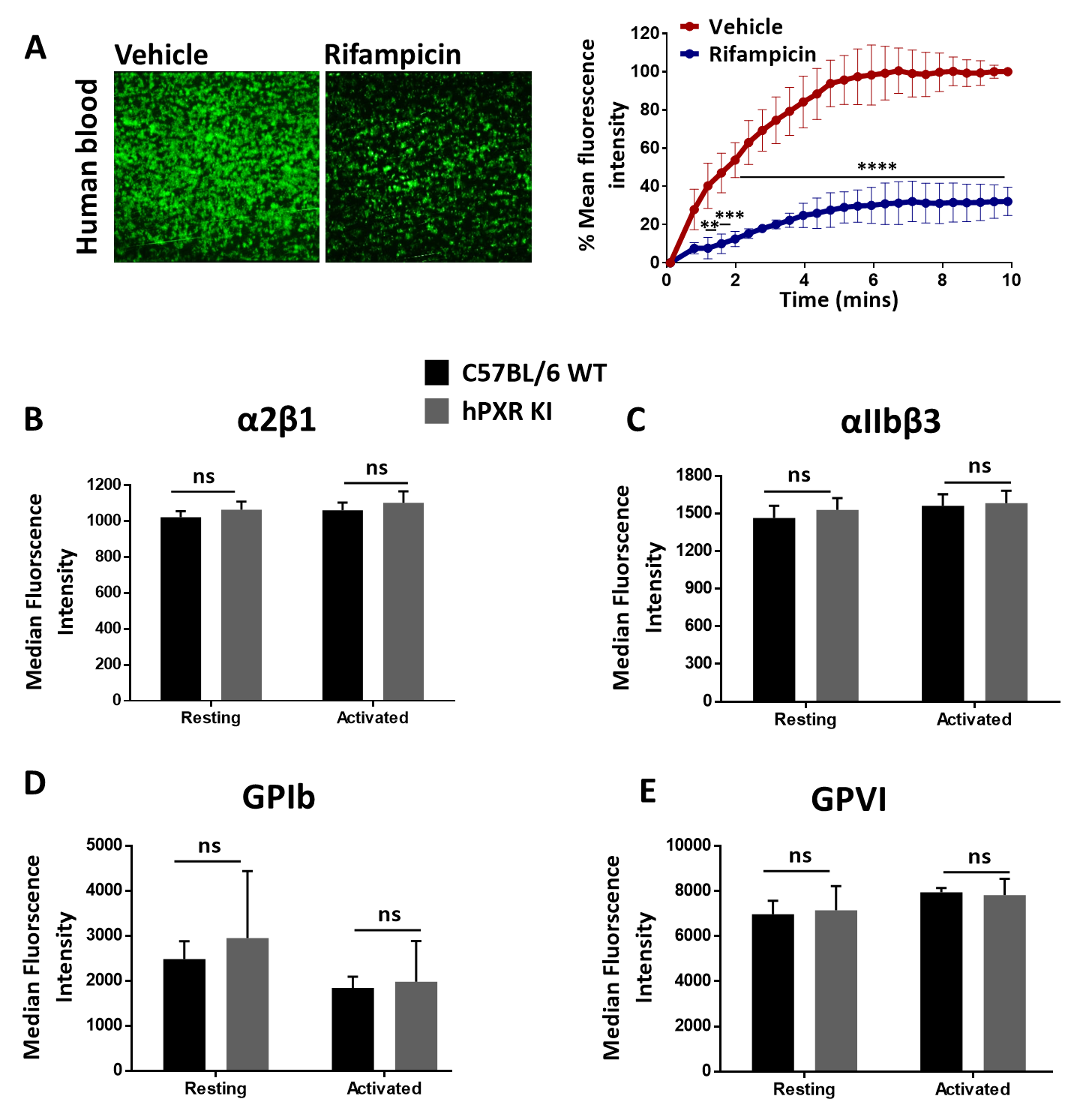
**

**Suppl. figure 3. PXR ligands inhibit thrombus formation *in vitro*.** **(A)** Human blood incubated with DiOC_6_ (5 μM) was perfused through collagen-coated (100 μg/ml) microfluidic chips at an arterial flow rate (20 dyne/cm^2^) after treatment with rifampicin or vehicle-control (DMSO 0.1% v/v) for 20 minutes. Representative images display thrombus formation. Quantified data represent mean thrombus fluorescence intensity normalised to fluorescence level of the vehicle-treated sample obtained at the end of the assay. The expression levels of **(B)** α2β1, **(C)** αIIbβ3, **(D)** GPIb, and **(E)** GPVI were analysed on resting and CRP-XL-stimulated (1 μg/ml) platelets from hPXR and C57BL/6 wild-type mice by flow cytometry. Data represent mean±SD (n≥3) where **P ≤ 0.01, ***P˂0.001 and ****P˂0.0001 was determined by two-way ANOVA (*in vitro* thrombus formation) and Student t-test (flow cytometry). Figure adapted from corresponding PhD thesis - Non-genomic effects of the Pregnane X Receptor (PXR) and Retinoid X Receptor (RXR) in platelets^49^.

**Suppl. Figure 4**

**
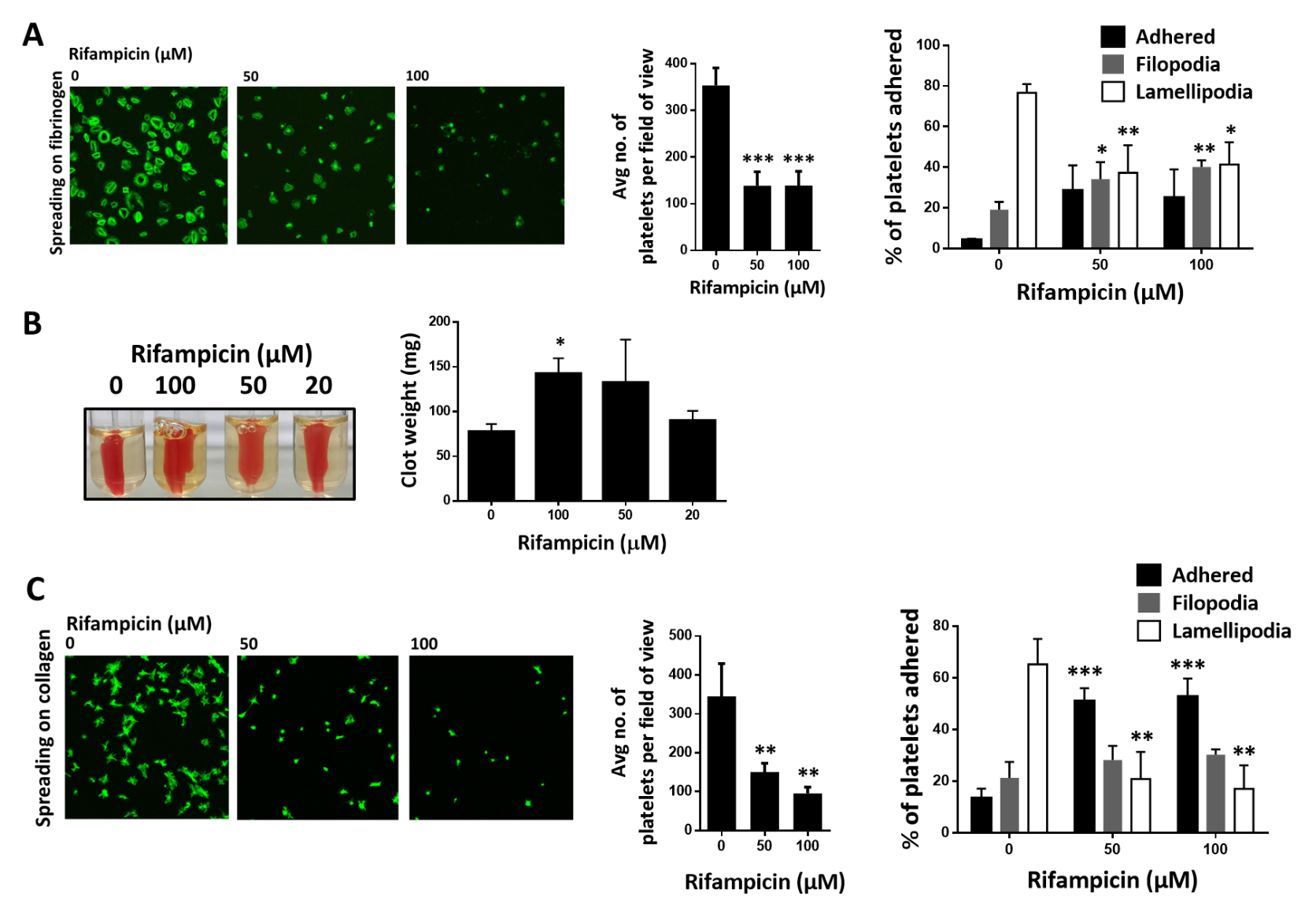
**

**Suppl. figure 4. Rifampicin inhibit outside-in signalling in platelets.** Human washed platelets (2x10^7^ cells/ml) were treated with rifampicin (50 and 100 μM) or vehicle-control (DMSO 0.1% v/v) for 20 minutes and added onto **(A)** fibrinogen (100 μg/ml) or **(C)** collagen-coated coverslips for 45 mins. Platelets were stained using Alexa-Fluor 488 for visualisation using a Nikon A1-R confocal microscope (100X). 5 images were captured of each sample at random locations. Representative images of platelet adhesion and spreading are shown. Cumulative data of platelets adhered in each sample is shown. Spreading platelets were divided into 3 classes: (adhered but not spread; filopodia: spreading platelets and lamellipodia: fully spread). Results expressed (as relative frequency) as the percentage of the total number of platelets adhered. **(B)** Human PRP was incubated with SR12813 (20, 50 and 100 μM) or vehicle-control (DMSO 0.1% v/v) for 20 minutes. Extent of clot retraction was determined by comparing clot weight after 60 minutes. Representative image of clot retraction after the end of the assay is shown. Cumulative data represent clot weight (in mg) of samples treated with SR12813 compared with vehicle-control. Data represent mean±SD (n≥3), *P˂0.05, **P˂0.01 and ***P˂0.001 was calculated by one-way ANOVA. Figure adapted from corresponding PhD thesis - Non-genomic effects of the Pregnane X Receptor (PXR) and Retinoid X Receptor (RXR) in platelets^49^.

**Suppl. Figure 5**

**
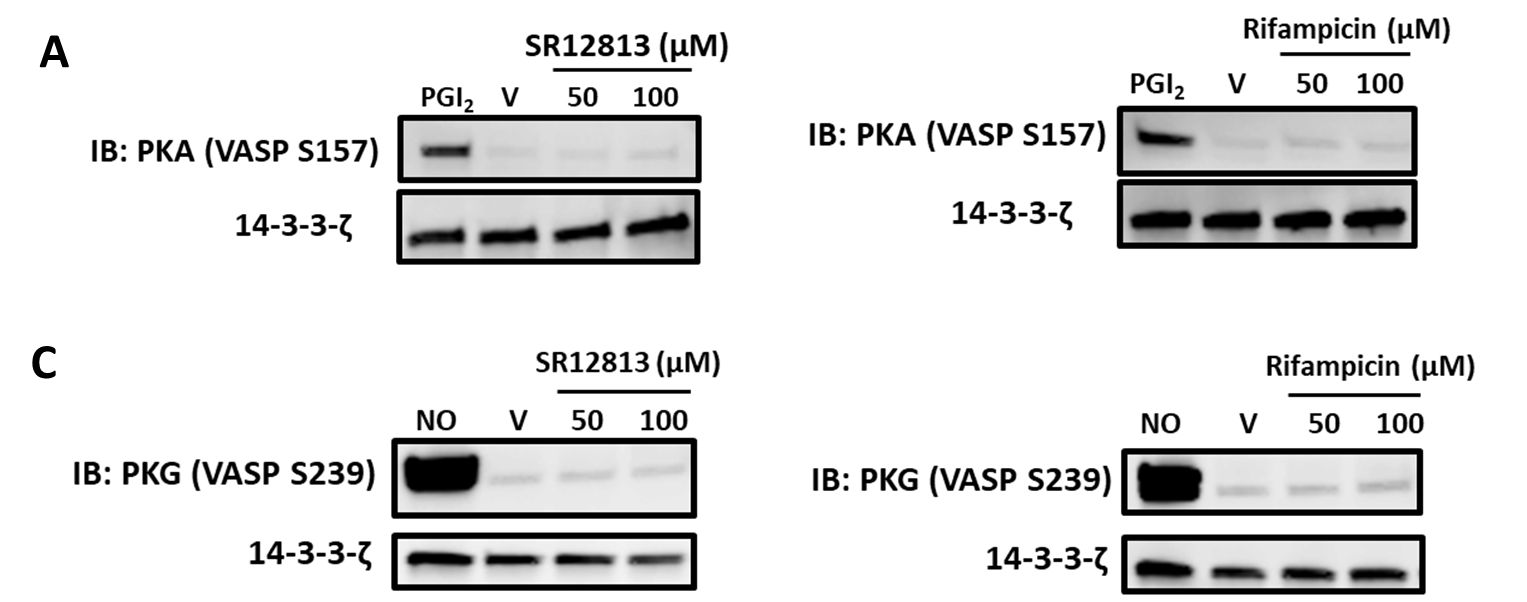
**

**Suppl. figure 5. Human PXR ligands does not regulate cyclic nucleotide mediated signalling in platelets.** Human washed platelets (4x10^8^ cells/ml) were tested for **(A)** VASP S157 and **(B)** VASP S239 phosphorylation in samples treated with SR12813 or rifampicin (0, 50 and 100 μM) or vehicle (DMSO 0.1% v/v) for 20 min. PGI_2_ and PAPANOATE (NO donor), which upregulates the activity of PKA and PKG were included as positive controls. 14-3-3-ζ was used as a loading control. Full length blots are shown in supplementary figure 11. Representative blots from 3 different experiments are shown. Figure adapted from corresponding PhD thesis - Non-genomic effects of the Pregnane X Receptor (PXR) and Retinoid X Receptor (RXR) in platelets^49^.

**Suppl. Figure 6**

**
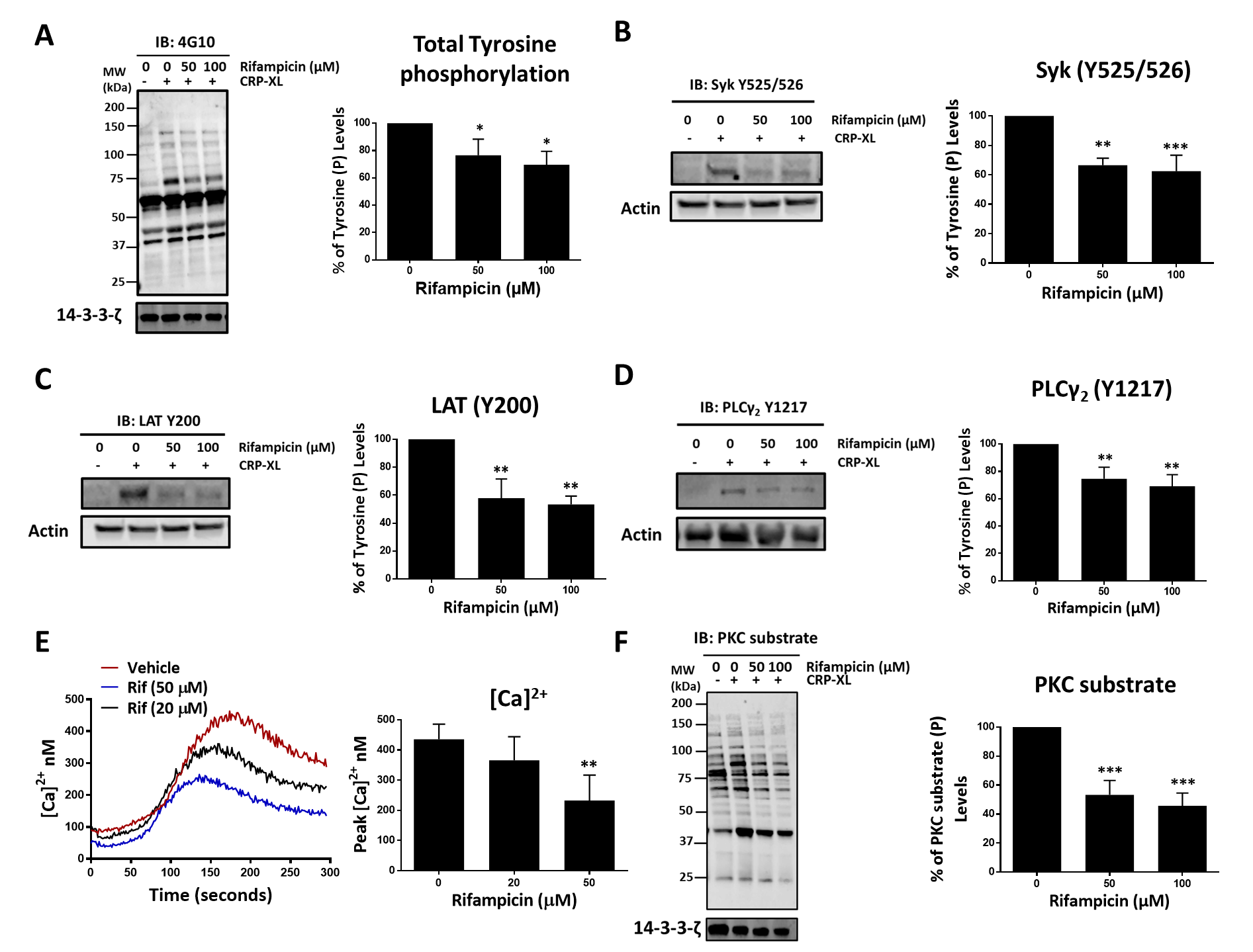
**

**Suppl. figure 6. Rifampicin negatively regulates GPVI-mediated signalling.** Platelets (4x10^8^ cells/ml) were pre-treated with vehicle (DMSO 0.1% v/v) or rifampicin (0, 50 and 100 μM) for 20 minutes and stimulated with CRP-XL (1 μg/ml) for 90 seconds in the presence of indomethacin (20 μM), cangrelor (1 μM), MRS2179 (100 μM) and EGTA (1 mM). Samples were tested for **(A)** Total tyrosine, **(B)** Syk (Y525/526), (**C)** LAT (Y200), **(D)** PLCγ2 (Y1217) and **(F)** PKC substrate phosphorylation. Representative immunoblots are shown. Levels of phosphorylation were quantified and expressed as a percentage of untreated (vehicle) controls. 14-3-3-ζ or actin was used as a loading control. Full length blots are shown in supplementary figure 9 **(E)** Calcium mobilisation was evaluated in Fura-2AM loaded platelets (4x10^8^ cells/ml) incubated with rifampicin (50 and 100 μM) or vehicle-control (DMSO 0.1% v/v) for 20 min prior to stimulation with CRP-XL (0.25 μg/ml). Traces of CRP-XL-stimulated calcium mobilisation over a period of 5 minutes. Cumulative data (peak calcium levels) of calcium mobilisation. Data represent mean±SD (n≥3) where *P˂0.05, **P˂0.01 and ***P˂0.001was determined by one-way ANOVA. Figure adapted from corresponding PhD thesis - Non-genomic effects of the Pregnane X Receptor (PXR) and Retinoid X Receptor (RXR) in platelets^49^.

**Suppl. figure 7**

**
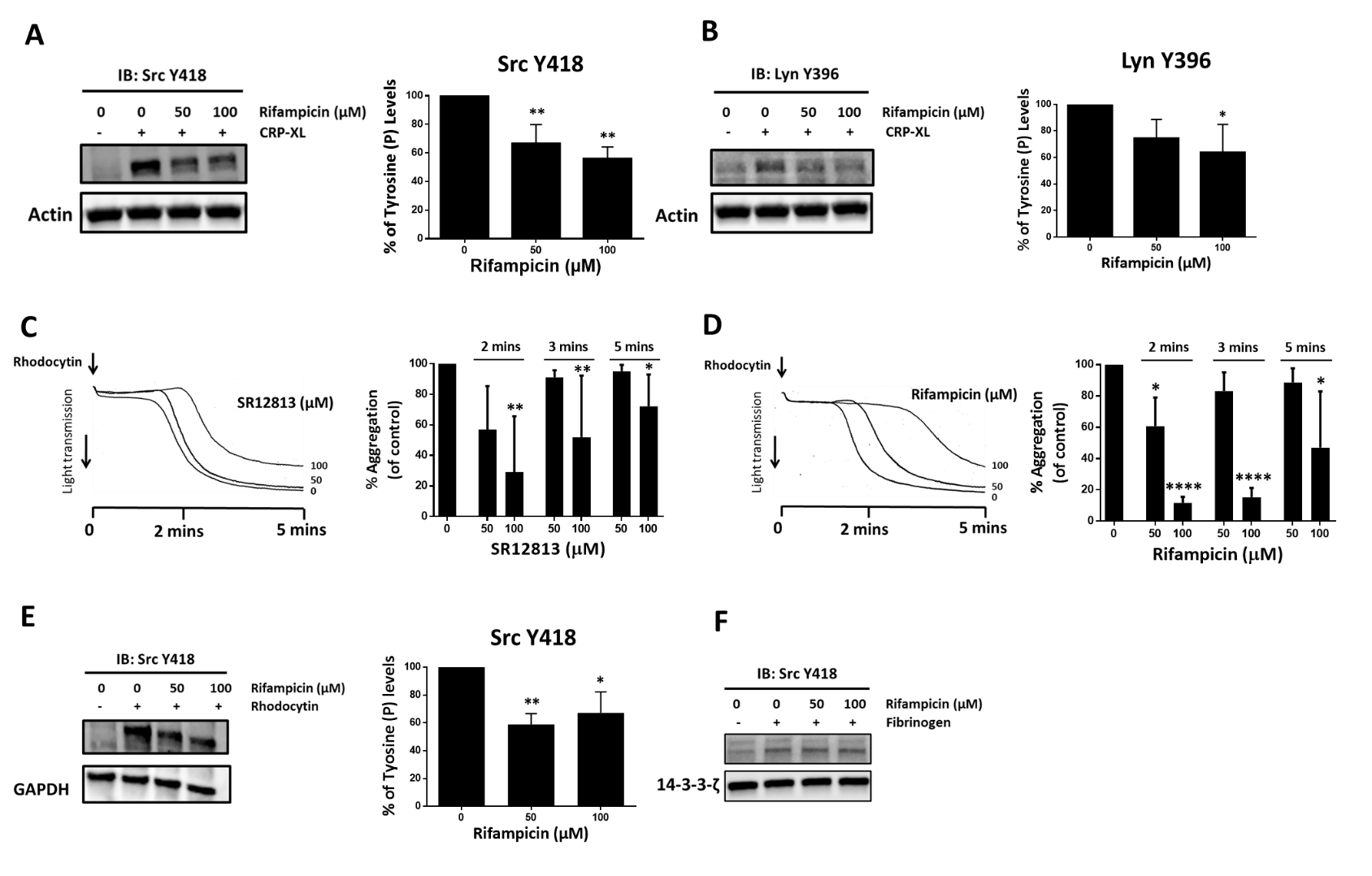
**

**Suppl. figure 7. Rifampicin inhibit tyrosine phosphorylation of SFKs.** Washed platelets (4x10^8^ cells/ml) were pre-treated with vehicle-control (DMSO 0.1% v/v) or rifampicin (0, 50 and 100 μM) for 20 minutes and stimulated for **(A, B)** 90 seconds with CRP-XL (1 μg/ml) or **(E)** 120 seconds with rhodocytin (100 nM) in the presence of indomethacin (20 μM), cangrelor (1 μM), MRS2179 (100 μM) and EGTA (1 mM). **(F)** Washed platelets (4x10^8^ cells/ml), pre-treated with rifampicin (0, 50 and 100 μM) or vehicle-control were exposed to fibrinogen-coated wells (100 μg/ml) of a tissue culture plate and allowed to adhere for 30 minutes. The samples were tested for Src (Y418) or Lyn (Y396) phosphorylation. Representative blots for the phosphorylation levels are shown. The phosphorylation levels were quantified and expressed as a percentage of untreated (vehicle) controls. Actin was used as a loading control. **(C, D)** Rhodocytin (100 nM) induced platelet aggregation was measured in human washed platelets (4x10^8^ cells/ml) pre-treated with SR12813 or rifampicin or vehicle-control (DMSO, 0.1% v/v). Aggregation was measured for 300 seconds. Representative aggregation traces are shown. Quantified data displays the percentage of aggregation of SR12813 or rifampicin treated samples (vehicle-treated samples represent 100% aggregation) at the end of 2 minutes, 3 minutes and 5 minutes. Full length blots are shown in supplementary figure 9 and supplementary figure 10. Results are mean±SD (n≥3), *P˂0.05, **P˂0.01, ***P˂0.001 and ****P˂0.0001 was calculated by one-way ANOVA. Figure adapted from corresponding PhD thesis - Non-genomic effects of the Pregnane X Receptor (PXR) and Retinoid X Receptor (RXR) in platelets^49^.

**Suppl. figure 8**

**
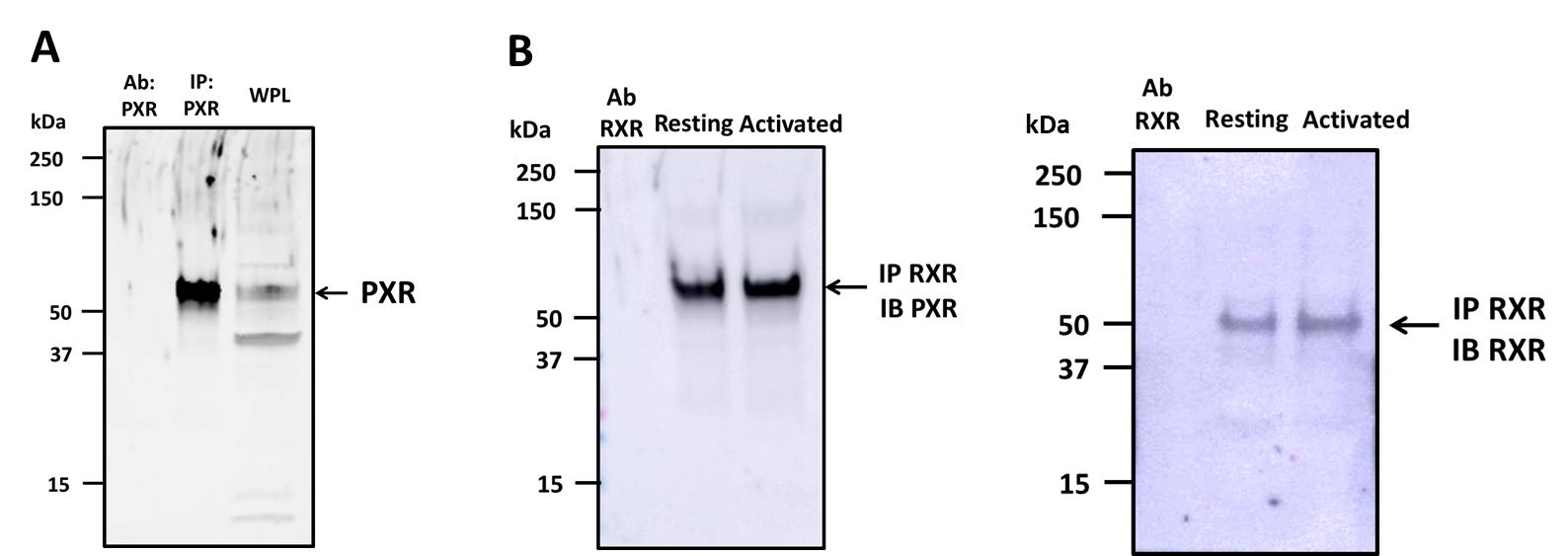
**

**Suppl. figure 8. (A)** Full blot image of figure 1A. PXR was immunoprecipitated (IP: PXR) from human platelets (IP: PXR), which was followed with a western blot analysis. Human whole platelet lysates (WPL) and antibody used to IP (Ab: PXR) PXR were used as positive and negative controls, respectively. **(Bi, Bii)** Full blot image of figure 2A. RXR was immunoprecipitated from human washed platelets. Immunoblot analysis was followed with the addition of an anti-PXR antibody and its detection using a secondary antibody that does not recognize denatured IgG. The presence of RXR was also confirmed in the same samples.

**Suppl. figure 9**

**
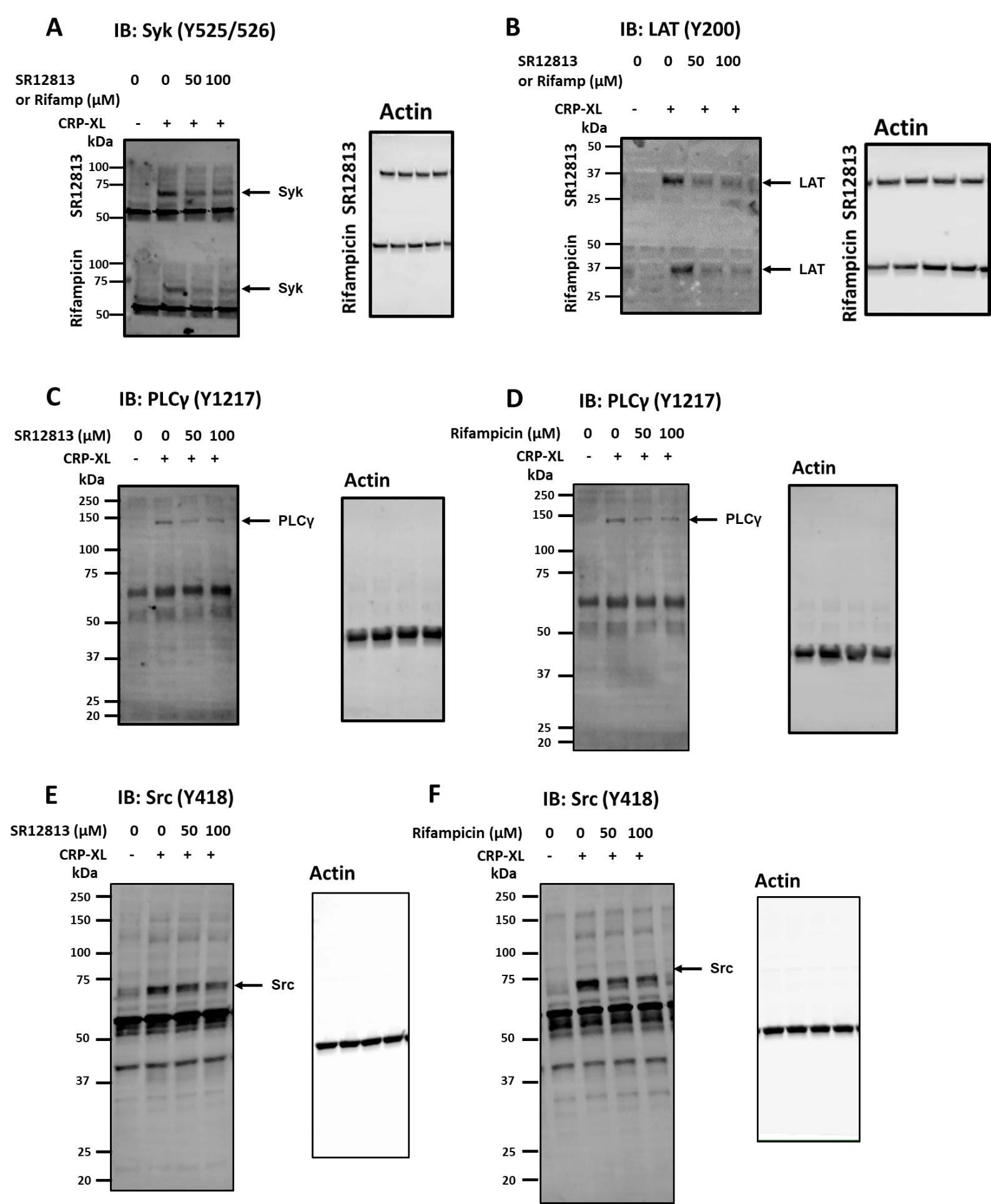
**

**Suppl. figure 9.** Platelets were pre-treated with vehicle (DMSO 0.1% v/v) or SR12813 (0, 50 and 100 μM) for 20 minutes and stimulated with CRP-XL (1 μg/ml) for 90 seconds in the presence of indomethacin (20 μM), cangrelor (1 μM), MRS2179 (100 μM) and EGTA (1 mM). Samples were tested for Syk (Y525/526), LAT (Y200), PLCγ2 (Y1217) and Src. **(A)** Full blot image of figure 7B and supplementary figure 6B. **(B)** Full blot image of figure 7C and supplementary figure 6C. **(C)** Full blot image of figure 7D. **(D)** Full blot image of supplementary figure 6D. **(E)** Full blot image of figure 8A. **(F)** Full blot image of supplementary figure 7A. Blots were reprobed with actin antibody to verify equivalent levels of protein loading.

**Suppl. figure 10**

**
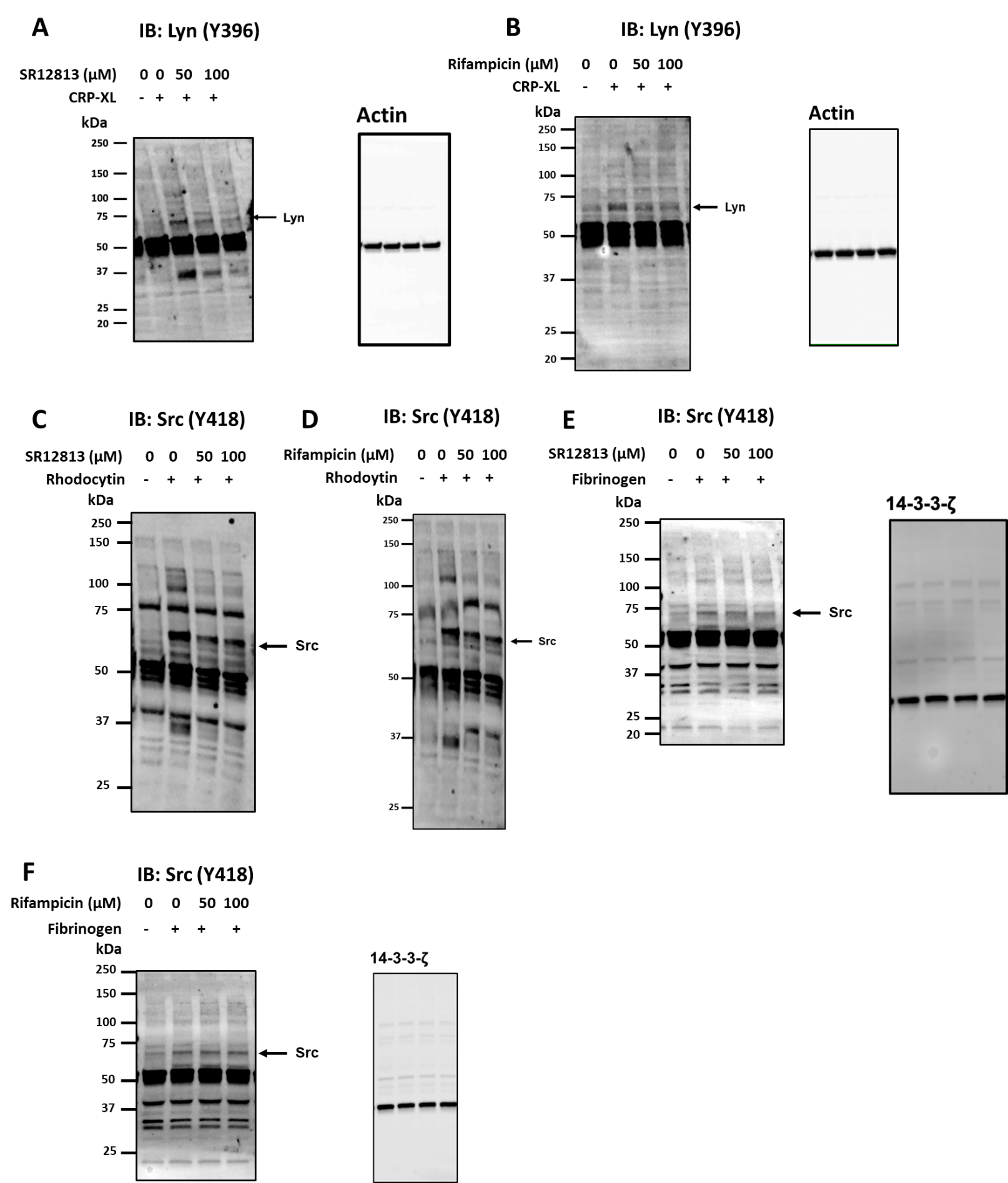
**

**Suppl. figure 10.** Platelets were pre-treated with vehicle-control (DMSO 0.1% v/v) or SR12813 (0, 50 and 100 μM) for 20 minutes and stimulated for 90 seconds with CRP-XL (1 μg/ml) or 120 seconds with rhodocytin (100 nM) in the presence of indomethacin (20 μM), cangrelor (1 μM), MRS2179 (100 μM) and EGTA (1 mM). Samples were tested for Lyn (Y396), or Src (Y418). **(A)** Full blot image of figure 8B. **(B)** Full blot image of supplementary figure 7B. **(C)** Full blot image of figure 8C. **(D)** Full blot image of supplementary figure 7E. Washed platelets, pre-treated with SR12813 (0, 50 and 100 μM) or vehicle-control were exposed to fibrinogen-coated wells (100 μg/ml) of a tissue culture plate and allowed to adhere for 30 minutes. Samples were tested for Src (Y418) phosphorylation. **(E)** Full blot image of figure 8D. **(F)** Full blot image of supplementary figure 7F. Blots were reprobed with actin or 14-3-3-ζ antibody to verify equivalent levels of protein loading.

**Suppl. figure 11**


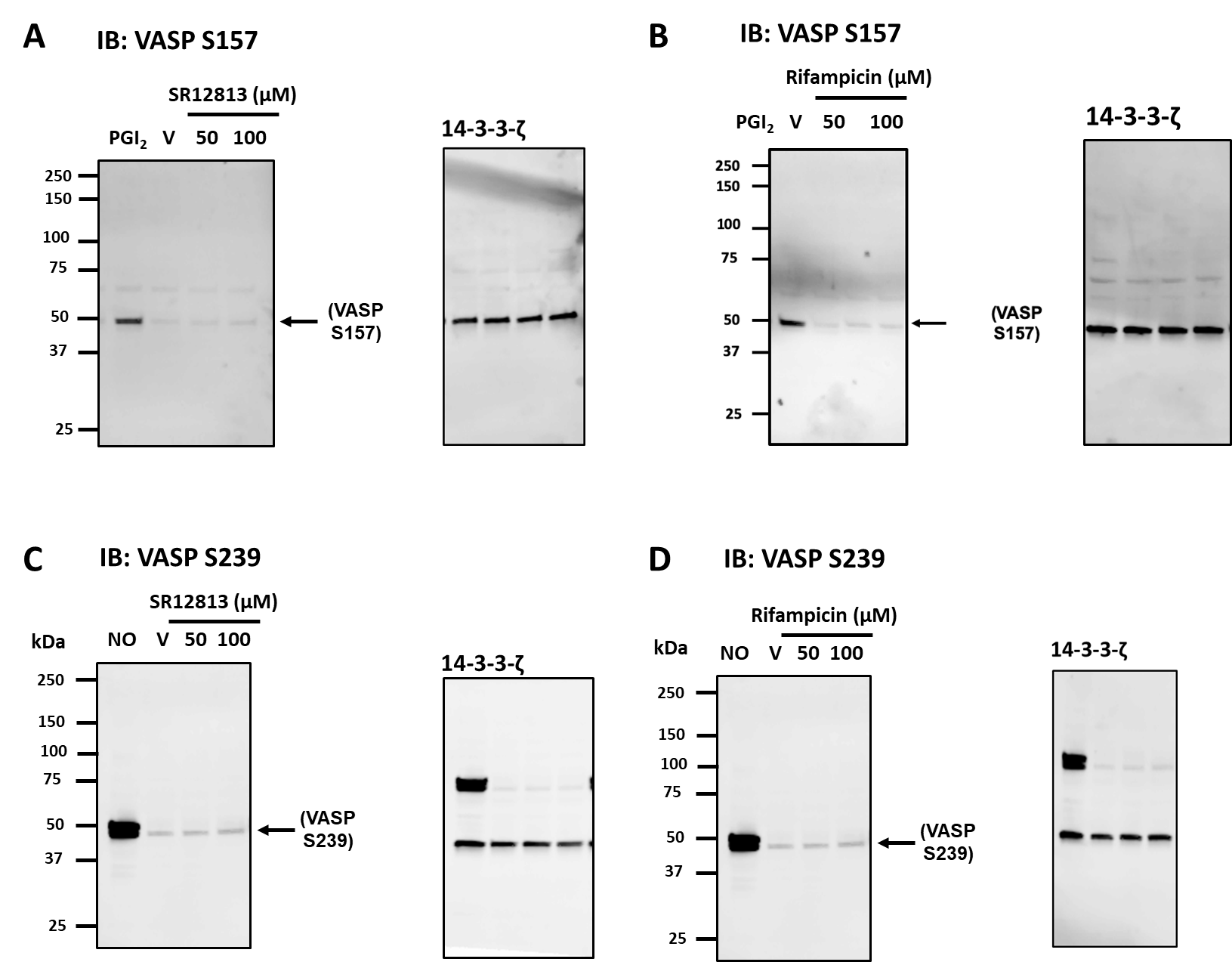


**Suppl. figure 11.** Human washed platelets were tested for VASP S157 and VASP S239 phosphorylation in samples treated with SR12813 or rifampicin (0, 50 and 100 μM) or vehicle (DMSO 0.1% v/v) for 20 min. PGI_2_ and PAPANOATE (NO donor), which upregulates the activity of PKA and PKG were included as positive controls. **(A)** Full blot image of supplementary figure 5A. **(B)** Full blot image of supplementary figure 5B. **(C)** Full blot image of supplementary figure 5C. **(D)** Full blot image of supplementary figure 5D. Blots were reprobed with 14-3-3-ζ antibody to verify equivalent levels of protein loading.
